# Reporting of the harms from randomized controlled trials for psoriasis: a cross-sectional meta-epidemiological study

**DOI:** 10.3389/fmed.2026.1734221

**Published:** 2026-02-27

**Authors:** Zhaoxin Yang, Yalan Xiong, Yi Shen, Zilin Cheng, Yangyingqi Dai, Pingsheng Hao, Zhipeng Hu

**Affiliations:** Hospital of Chengdu University of Traditional Chinese Medicine, Chengdu, China

**Keywords:** a methodologicalstudy, adverse events, CONSORT-harms, psoriasis, randomized controlled trials

## Abstract

**Objectives:**

Analysis of adverse events (AEs) in randomized controlled trials (RCTs) related to psoriasis.

**Design:**

A cross-sectional meta-epidemiological study.

**Data sources:**

We conducted a comprehensive search of PubMed and the Cochrane Database for studies meeting our eligibility criteria from January 2020 to July 2025.

**Eligibility criteria:**

RCTs specifically investigating non-articular psoriasis were included. All long-term extension (LTE) studies were excluded; however, psoriasis-related RCTs utilizing both double-blind and open-label designs were incorporated.

**Main outcome measures:**

We assessed (1) general characteristics of psoriasis RCTs; (2) the Adverse Event Reporting Completeness Index (AERCI) Score for reporting completeness; (3) specific details of AEs; (4) factors associated with reporting completeness.

**Results:**

A total of 187 psoriasis RCTs published between 2020 and 2025 were included. The median AERCI-Core score was 6.00 (IQR: 2.00–8.00), indicating suboptimal overall reporting quality, with only 26.2% of studies rated as high quality. Adherence to individual reporting items varied widely, with particularly low rates observed for AE management measures (16.0%), timing of AE onset (20.3%), outcome/resolution (26.7%), and use of standardized coding systems (31.6%). Multivariable linear regression identified journal impact factor [β = 0.27, 95% CI (0.13, 0.40), *P* < 0.001] and pustular psoriasis subtype [β = 1.85, 95% CI (0.10, 3.60), P = 0.038] as independent predictors of higher reporting completeness.

**Conclusions:**

This study identified inadequate reporting of adverse events in RCTs for non-articular psoriasis. After adjusting for confounding variables, a higher journal impact factor and a focus on pustular psoriasis were positively associated with the complete reporting of AEs. To enhance the utility of safety data for clinical decision-making, future trials should rigorously implement the Consolidated Standards of Reporting Trials—Harms 2022 (CONSORT-Harms 2022) recommendations, thereby providing patients with more balanced information on the benefits and harms of psoriasis therapies.

## 1 Introduction

Psoriasis is a chronic, systemic inflammatory skin disease affecting over 60 million individuals worldwide. It is frequently associated with multiple comorbidities and imposes a substantial burden on patients' quality of life (1–3). In 2014, the World Health Organization (WHO) formally recognized psoriasis as a non-communicable, disabling, and currently incurable disease, explicitly identifying its global burden as a priority research area (1). In recent years, biologics targeting pathways such as IL-17 and IL-23 have become first-line therapies for moderate-to-severe plaque psoriasis, with their efficacy well-established (4). Nevertheless, the safety profiles vary among different biologics, and evidence on their long-term safety remains insufficient (5). Within this con-text, clinical decision-making necessitates a more cautious balance between therapeutic benefits and potential risks.

RCTs, considered the gold standard for establishing causal effects of interventions, provide an ideal framework for the systematic collection of safety data through their prospective, randomized, and controlled design (6, 7). Compared with post-marketing surveillance systems, RCTs enable earlier and more precise monitoring and assessment of potential drug risks (8). Nonetheless, despite the methodological capacity of RCTs to yield high-quality safety data, the actual reporting quality has remained suboptimal (9). Reviews across multiple therapeutic fields have confirmed widespread deficiencies in the application of reporting guidelines such as CONSORT-Harms, which substantially limits the value of this data for clinical safety assessment. This reflects a persistent and significant gap between reporting standards and actual practice (10–13).

In the field of psoriasis treatment, the widespread use of biologics and small-molecule agents has heightened concerns regarding treatment safety (4, 14). Although the number of relevant RCTs has grown substantially, systematic assessments of the quality of their safety reporting remain scarce. A study by Beytout et al. (14) has recently addressed this gap by evaluating the reporting quality of RCTs on systemic psoriasis therapies initiated after September 2009, revealing significant inconsistencies between published articles and the corresponding ClinicalTrials.gov registry entries. However, this study has several limitations. First, its evaluation was based on the 2004 CONSORT-Harms checklist and did not incorporate the formally updated CONSORT-Harms 2022 standards, with the data cutoff being October 2022. Consequently, it cannot reflect current reporting practices or potential improvements following the dissemination of the new guidelines. Second, the analysis remained primarily descriptive and did not employ multivariate models to investigate the potential factors influencing reporting quality, thereby limiting a deeper understanding of the root causes of these deficiencies.

Therefore, as a necessary extension and complement to the existing evidence, this study aims: First, to expand the analytical scope to include RCTs investigating all categories of interventions for psoriasis published between 2020 and 2025, thereby capturing a more comprehensive overview of the contemporary clinical research landscape. Second, to establish a structured assessment framework based on the latest CONSORT-Harms 2022 guidelines (15), enabling a granular evaluation of adverse event reporting across its entire workflow—from collection and analysis to presentation. Third, moving beyond a descriptive account of current reporting practices, we will, for the first time, employ multivariable analysis to investigate potential associations between the completeness of adverse event reporting and factors such as journal characteristics and trial design features. This study seeks to elucidate novel deficiencies in current reporting practices and to provide an empirical foundation for enhancing the transparency and utility of future RCTs in this field.

## 2 Materials and methods

We conducted a cross-sectional meta-epidemiological study to systematically analyze the general characteristics and reporting status of AEs in psoriasis RCTs published between January 2020 and July 2025. The methodology for this study was pre-specified and registered via the Open Science Framework (OSF)^1^; any deviations from the protocol are accessible on the OSF platform. This study was conducted and reported in accordance with the reporting guidelines for meta-epidemiological research.

### 2.1 Patient and public involvement

Patients and/or the public were not involved in the design, conduct, reporting, or dissemination plans of this research.

### 2.2 Identification and selection of articles

This review focused on psoriasis RCTs published in the 96 dermatology journals recognized by the 2025 Journal Citation Reports. The selection of PubMed and the Cochrane Database was based on their comprehensive coverage of the relevant dermatology literature. We therefore searched these two databases for studies published between January 2020 and July 2025. The detailed search strategy and journal list are provided in the online Supplementary material.

We used EndNote 21 to screen and exclude studies. Two investigators (ZX and YL) independently screened the titles and abstracts based on predetermined eligibility criteria. This was followed by independent full-text reviews conducted by two additional researchers (ZL and YQ). Discrepancies during the screening process were re-solved through consensus discussions between two senior investigators (ZP and PS). The study selection process is detailed in the PRISMA flow diagram. For the purpose of screening, a record was considered “full text unavailable” (and thus excluded) if, after exhaustive efforts, the complete manuscript could not be retrieved. Primary reasons for unavailability were: (1) paywall restrictions not covered by our institutional subscriptions; (2) database limitations providing only an abstract without a full-text link; and (3) unavailability after direct requests to corresponding authors. Our search was conducted in English databases without language filters.

### 2.3 Inclusion criteria

Eligibility criteria for RCT inclusion, followed the population, intervention, comparator, outcome, study type (PICOS) framework.

Population: Patients with various forms of psoriasis (plaque, erythrodermic, pustular), excluding psoriatic arthritis, as psoriatic arthritis represents a distinct rheumatic immune disease requiring fundamentally different efficacy assessment approaches from cutaneous psoriasis.

Interventions: Any medical intervention (pharmacological/non-pharmacological) for treating or preventing non-articular psoriasis.

Comparator(s): Any

Outcome(s): All outcomes measuring treatment safety in psoriasis were included without restriction.

Study type: 1. Original randomized controlled trials.

2. Studies excluded non-randomized investigations (e.g., observational studies, single-arm trials), secondary analyses of RCTs, and extensions including but not limited to long-term extension studies, post-hoc analyses, pre-specified or non-pre-specified subgroup analyses, pooled analyses, meta-analyses, and patient-level data reanalyses.

### 2.4 Data extraction

Data extraction was performed independently by two investigators (ZX and YL) using a standardized form pre-designed in Microsoft Office Excel. The extracted information encompassed study characteristics and variables related to AE reporting.

The extracted study characteristics included publication year, journal impact factor, funding source, sample size, disease subtype, and center type. To comprehensively characterize adverse event reporting practices, we extracted data for 20 reporting items aligned with the CONSORT-Harms 2022 guidelines. These items encompass key domains of AE collection, analysis, and presentation, including assessor identity, confirmation methods, timing, severity grading, management measures, outcomes, statistical analysis, pre-specification, and coding systems (See Supplementary Table S1).

All data were extracted from the main text and Supplementary material of each included article. Any discrepancies between reviewers were resolved through discussion to reach consensus, with arbitration by a senior investigator (SY) when necessary.

### 2.5 Assessment of adverse event reporting completeness

To mitigate potential conceptual overlap between individual AE reporting items and the study outcomes, a composite index was constructed to quantify the overall completeness of AE reporting.

The primary outcome measure was the Adverse Event Reporting Completeness Index–Core (AERCI-Core). This index comprises 12 specific harm items, selected based on explicit reviewer endorsements and their alignment with the core elements of the CONSORT-Harms checklist. These items encompass the fundamental components of adverse event reporting, including: identification of the AE assessor(s), methods for AE confirmation, time of onset, severity grading, management measures, outcome or resolution, discontinuations due to AEs, explicit reporting of serious AE types, reporting of adverse events of special interest, between-group statistical comparison, pre-specification of harm outcomes in the study protocol or trial registry, and the use of a standardized AE coding system (e.g., MedDRA or WHO-ART)(See items 1–12 in Supplementary Table S1).

Additionally, an extended index (AERCI-Extended) was developed. This index incorporates eight supplementary items recommended by the CONSORT-Harms 2022 statement and was employed specifically in sensitivity analyses to assess the robustness of the findings under varying definitions of reporting completeness (See items 13–20 in Supplementary Table S1).

Each item was scored dichotomously (1 = adequately reported; 0 = not reported or unclearly reported). The total AERCI-Core score ranges from 0 to 12, and the AERCI-Extended score from 0 to 20, with higher scores reflecting greater completeness and transparency in adverse event reporting. The total AERCI-Core scores were categorized into three groups: low (≤the 25th percentile), moderate (interquartile range, between the 25th and 75th percentiles), and high (≥ the 75th percentile). All items were extracted independently by two investigators (ZX and YL) using a standardized data abstraction form. Discrepancies were resolved by a senior researcher (SY).

### 2.6 Covariates

Based on prior literature and *a priori* hypotheses, the following study-level characteristics were evaluated as potential predictors of AE reporting completeness: funding source (industry, institutional, mixed, personal, or none), disease subtype (plaque psoriasis, psoriasis in special areas, pustular psoriasis), journal impact factor (continuous variable), publication year (continuous variable), and sample size (continuous variable). These variables were selected as they may plausibly influence trial design, safety monitoring procedures, and reporting practices.

### 2.7 Data analysis

To assess whether the publication of the CONSORT-Harms 2022 guidelines influenced researcher adherence to its items, a subgroup analysis was conducted to compare the rates of item adherence in RCT reports published before vs. after the guideline release. Specifically, RCTs were dichotomized into two periods based on their publication date relative to the release of the CONSORT-Harms 2022 statement: the pre-guideline period (studies published 2020–2022) and the post-guideline period (studies published 2023–2025). For each reporting item, the proportion of studies that reported “yes” (i.e., fulfilled the item's requirement) was calculated separately for each period. Differences in these proportions were compared for statistical significance using the Chi-square test or Fisher's exact test, as appropriate. Categorical variables are summarized as frequency and percentage. The normality of continuous variables was assessed using the Shapiro–Wilk test. Normally distributed data are presented as mean ± standard deviation, while non-normally distributed data are presented as median (interquartile range).

The primary analysis employed multiple linear regression, with the AERCI-Core score as the dependent variable, to investigate associations between study characteristics and the completeness of core adverse event reporting. Initial univariable analyses were followed by the construction of a multivariable model incorporating all prespecified covariates. Results are reported as regression coefficients (β) with their corresponding 95% confidence intervals (CIs).

Multicollinearity among covariates was assessed using variance inflation factors (VIFs), with a VIF value ≥5 indicating potential collinearity concerns. No severe multicollinearity was detected. For categorical variables, the following reference categories were specified: “none” for funding source and “plaque psoriasis” for disease classification.

Sensitivity analyses were conducted to evaluate the robustness of the findings. First, the AERCI-Core score was categorized into tertiles (low, medium, high) and analyzed using ordinal logistic regression. Second, the primary multivariate analysis was repeated using the AERCI-Extended score as the outcome variable. Third, the analysis was repeated after excluding studies with a sample size below 30. The consistency in the direction and magnitude of associations across these analyses was considered evidence of robust results.

All statistical analyses were performed using R software (version 4.4.2). A two-sided *P*-value of < 0.05 was considered statistically significant.

## 3 Results

**Figure 1:**
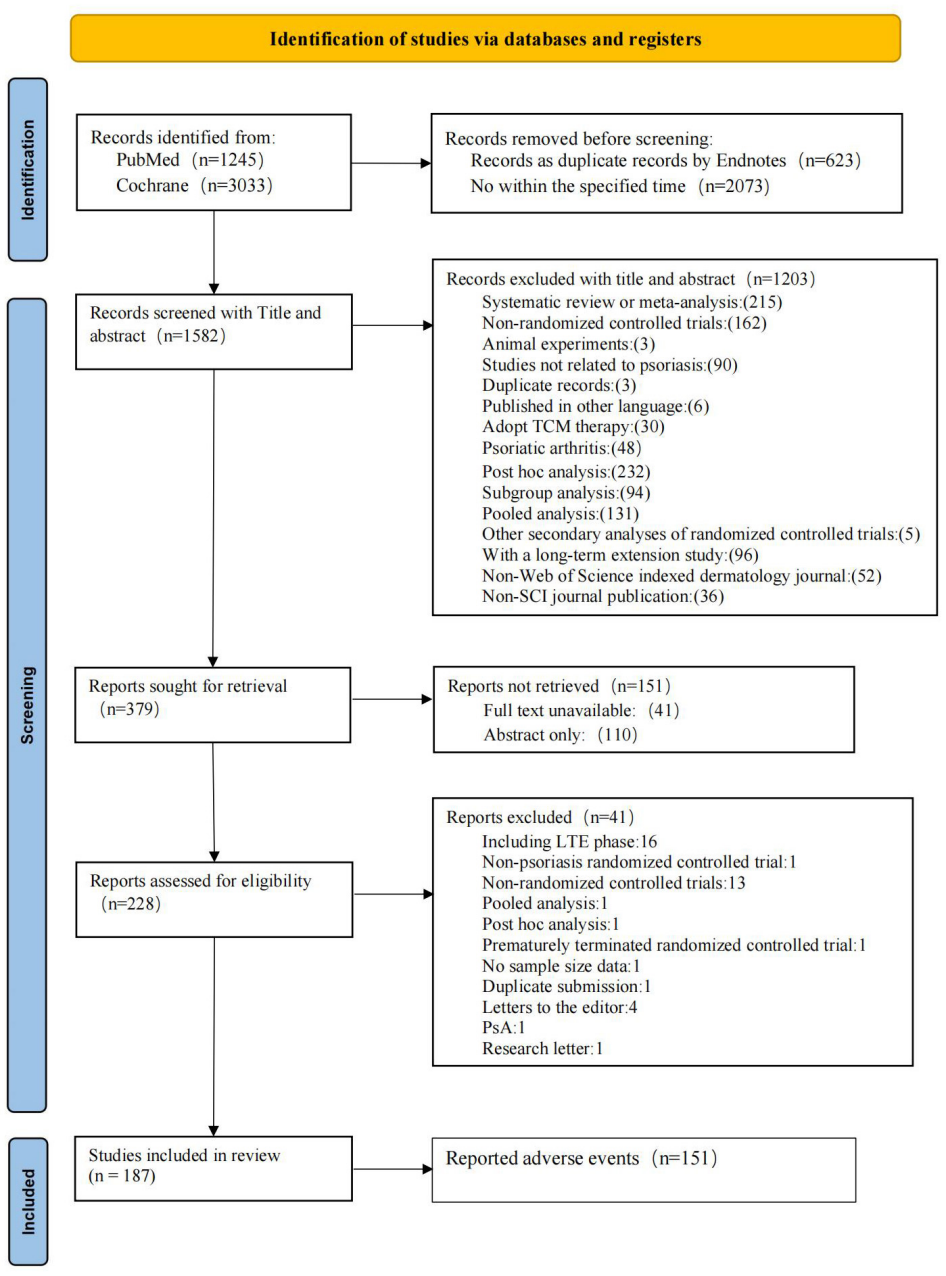
Flow chart of the study selection process. Abbreviations: PsA, Psoriatic Arthritis; TCM, Traditional Chinese Medicine; LTE, Long-Term Extension; SCI, Science Citation Index.

After removing duplicates and studies outside the specified time frame, 1,582 records were identified for screening. Following the initial screening, which excluded 1,203 articles (including systematic reviews, meta-analyses, and non-randomized con-trolled trials), 379 studies were selected for full-text review. Following the exclusion of 151 records lacking full text (including abstract only), 228 studies proceeded to the full-text assessment stage. After excluding 41 studies due to premature termination or inclusion of LTE phases, 187 RCTs ultimately met the inclusion criteria (Figure 1). The list of studies excluded after full-text review is provided in the Supplementary material.

### 3.1 General characteristics of eligibility studies

Table 1 presents the general characteristics of the included studies. The publication years were evenly distributed, with the highest number of studies published in 2024 (38 studies, 20.3%) and the lowest in 2025 (22 studies, 11.8%). Among the 187 studies, 110 (58.8%) were multicenter trials, 128 (68.5%) were published in JIF Q1 journals, and 136 (72.7%) reported a trial registration number. The mean impact factor was 5.89 (3.54). Institutional funding supported 133 studies (71.1%), and the majority of studies (105 studies, 56.2%) had no more than 10 authors. Regarding sample size distribution, 92 studies (49.2%) enrolled between 101 and 1,000 participants. Plaque psoriasis was the pre-dominant disease category (145 studies, 77.5%). In terms of control groups, 62 studies (33.2%) used a placebo control, while 79 studies (42.2%) employed an active comparator.

**Table 1:** General characteristics of the included studies^*^.

**Characteristic**	**No (%)**	**No (%)**
	**(*****N*** = **187)**	**Studies reported AEs**
**Publication year**		
2020	29 (15.5)	25 (16.3)
2021	36 (19.3)	32 (20.9)
2022	32 (17.1)	24 (15.7)
2023	30 (16.0)	26 (17.0)
2024	38 (20.3)	29 (19.0)
2025	22 (11.8)	17 (11.1)
**Type of study center**		
Single-center	74 (39.6)	53 (34.6)
Multi—center	110 (58.8)	98 (64.1)
Unreported	3 (1.6)	2 (1.3)
**JIF Quartile**		
Q1	128 (68.5)	110 (71.9)
Q2	27 (14.4)	21 (13.7)
Q3	32 (17.1)	22 (14.4)
Q4	0	0
**Registration number**		
Reported	136 (72.7)	114 (74.5)
Unreported	51 (27.3)	39 (25.5)
Journal impact factor, mean(SD)		5.89 (3.54)
**Funding** ^*^		
Institutional funding	133 (71.1)	108 (70.6)
Mixed funding	15 (8)	12 (7.8)
None	37 (19.8)	32 (20.9)
Unreported	1 (0.5)	0
Individual funding	1 (0.5)	1 (0.7)
**Number of authors** ^*^		
≤10	105 (56.2)	79 (51.6)
11–20	70 (37.4)	62 (40.5)
>20	12 (6.4)	12 (7.8)
**Sample size**		
≤30	32 (17.1)	21 (13.7)
31–100	57 (30.5)	47 (30.7)
101–1000	92 (49.2)	80 (52.3)
>1000	6 (3.2)	5 (3.3)
**Disease categories** ^*^		
Plaque psoriasis	145 (77.5)	116 (75.8)
Special area psoriasis	30 (16)	26 (17.0)
Pustular psoriasis	12 (6.4)	11 (7.2)
**Type of control group**		
Active	79 (42.2)	64 (41.8)
Placebo	62 (33.2)	54 (35.3)
Active + placebo	18 (9.6)	16 (10.5)
Others	28 (15)	19 (12.4)

^*^Values are presented as n (%); percentages are rounded and may not sum to 100.

**Figure 2:**
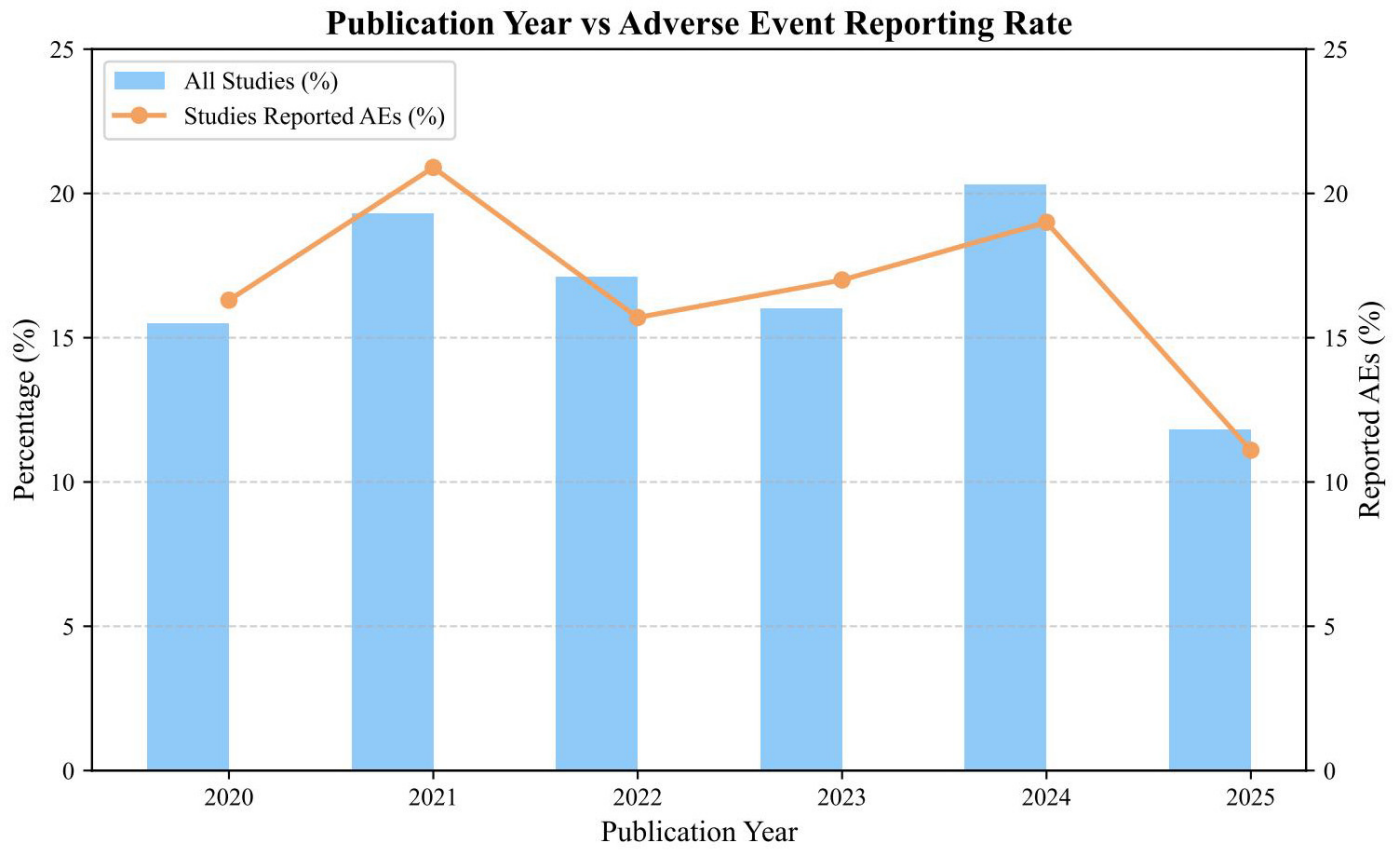
Annual distribution of included psoriasis randomized controlled trials and those reporting adverse events. This bar chart illustrates the yearly distribution of the 187 included randomized controlled trials (RCTs) from 2020 to 2025.

The relationship between the reporting rate of adverse events and the year of publication is presented in Figure 2.

### 3.2 Overall reporting quality and item adherence

#### 3.2.1 Distribution of AERCI-Core scores

The assessment based on the CONSORT-Harms 2022 guidelines indicates that the overall quality of adverse event reporting in psoriasis RCTs is suboptimal. Based on the CONSORT-Harms 2022 guidelines, the median AERCI-Core score was 6.00 (IQR: 2.00–8.00; total range 0–12), and the median AERCI-Extended score was 15.00 (IQR: 7.00–19.00; total range 0–20). According to the AERCI-Core scores, 49 studies (26.2%) were classified as high-quality (≥8 points), 88 (47.1%) as moderate-quality (2.00–8.00 points), and 50 (26.7%) as low-quality (≤2 points). Only three studies achieved a perfect score of 12 on the AERCI-Core.

#### 3.2.2 Reporting of individual adverse event items

**Figure 3:**
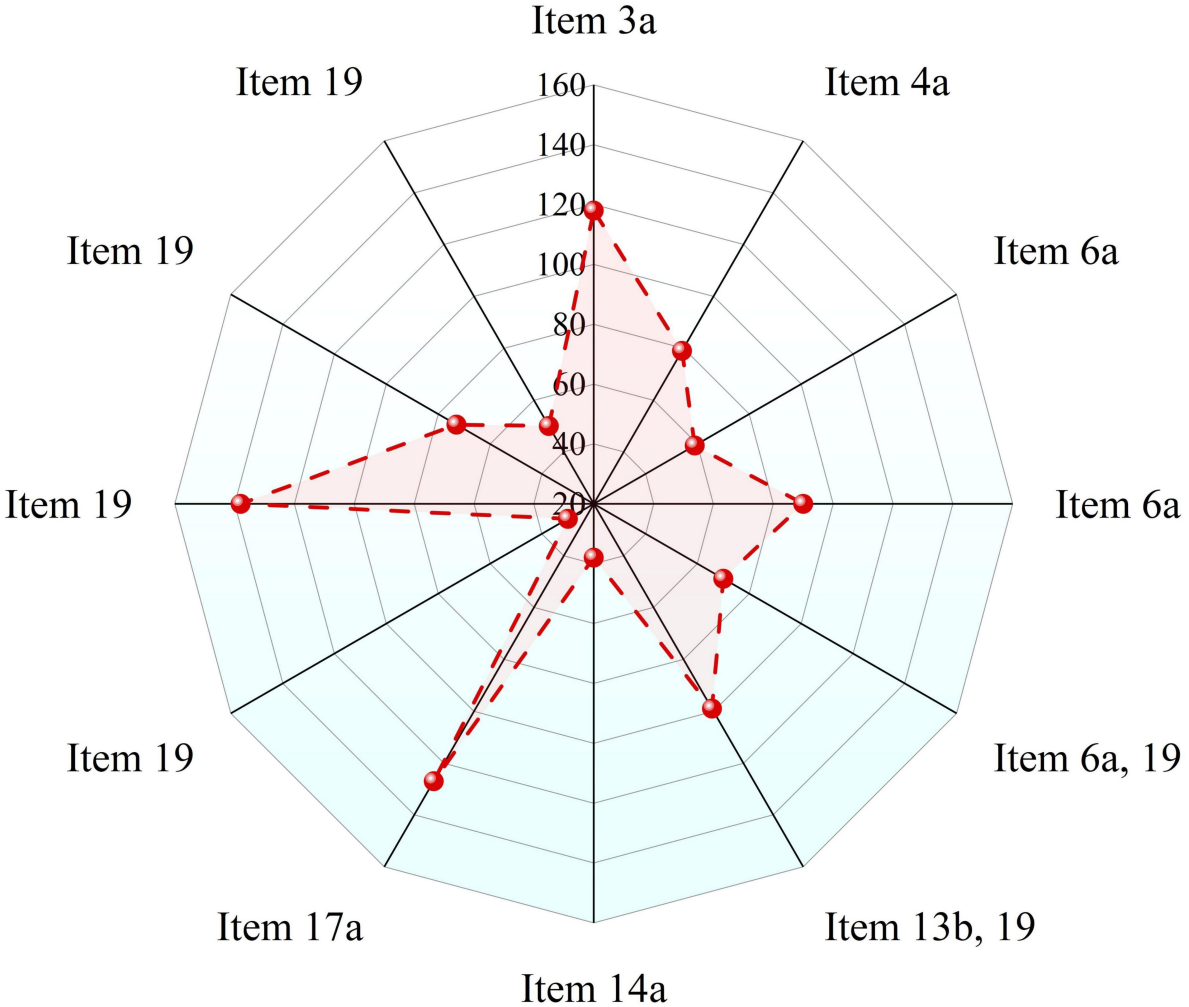
Frequency of compliance with the 12 AERCI Core items across 187 psoriasis randomized controlled trials. This bar chart displays the proportion of the 187 included RCTs that adequately reported each of the 12 core items of the Adverse Event Reporting Completeness Index (AERCI Core).

Each component of the AERCI was explicitly mapped to the CONSORT–Harms (2022) checklist, and the proportion of trials reporting each item is presented in Table 2 and Figure 3. Based on the CONSORT-Harms 2022 guidelines, we evaluated the compliance of the 187 psoriasis RCTs across the 12 core adverse event reporting items. Overall, substantial variability in item-level compliance was observed. The compliance rate was below 50% for the majority of items (8/12, 66.7%), indicating pronounced deficiencies across multiple key details of current adverse event reporting practices.

**Table 2:** Mapping of adverse event reporting to CONSORT-harms (2022)^*^.

**No**.	**CONSORT-harms (2022) item**	**CONSORT-harms item description**	**AERCI-core corresponding component**	**Trials reporting the item, *n* (%)**
1	Item 3a	How harms were identified (methods and definitions)	Definition and method of adverse event ascertainment	118(63.1%)
2	Item 4a	Eligibility criteria for participants	AE assessor identity	79 (42.2%)
3	Item 6a	Use of a coding system for harms (e.g., MedDRA, WHO-ART)	MedDRA/WHO-ART used	59 (31.6%)
4	Item 6a	Harm outcomes pre-specified in protocol/registry	Registry/protocol mention	90 (48.1%)
5	Item 6a, 19	Definition and reporting of adverse events of special interest	AESI specified	70 (37.4%)
6	Item 13b, 19	Participants withdrawn or lost to follow-up due to harms	AE-related withdrawal	99 (52.9%)
7	Item 14a	When harms were collected and the duration of follow-up	Time of onset reported	38 (20.3%)
8	Item 17a	Statistical methods used to compare harms between intervention groups	Between-group AE analysis	127 (67.9%)
9	Item 19	How harms were managed	AE management described	30 (16.0%)
10	Item 19	Severity grading of harms	AE severity reported	138 (73.8%)
11	Item 19	Numbers and types of serious adverse events	SAE types specified	73 (39.0%)
12	Item 19	Outcomes of harms (e.g., resolved, ongoing, sequelae)	AE outcome reported	50 (26.7%)

^*^Only CONSORT-Harms items directly related to adverse event collection, analysis, and reporting were included in the AERCI. Items addressing general trial design or reporting (e.g., randomization, allocation concealment, baseline characteristics) were not incorporated into the composite score.

^*^The full CONSORT-Harms 2022 checklist was used as the conceptual framework. A subset of harms-specific, operationalizable items was selected to construct the AERCI.

^*^This table provides the explicit mapping between the 12 core items of the AERCI-Core instrument and their corresponding items in the CONSORT-Harms (2022) checklist. The ‘Trials Reporting the Item, *n* (%)' column indicates the frequency and proportion of the 187 included RCTs that adequately reported each specific item.

Adherence rates varied substantially across the 12 core items. The most consistently reported item was Item 19 (Severity grading of adverse events), with an adherence rate of 73.8%. This was followed by Item 17a (Between-group statistical comparison of AEs) at 67.9% and Item 3a (Method(s) for identifying AEs) at 63.1%.

Items with lower adherence rates were predominantly those concerning methodological description and the detailed reporting of results. Notably, adherence was particularly poor for items detailing specific methodological and reporting elements. The lowest rates were observed for Item 19(Management measures for AEs) at 16.0% and Item 14a (Timing of AE occurrence) at 20.3%. Furthermore, adherence remained suboptimal for Item 19(Outcome/resolution of AEs) at 26.7% and Item 6a (Use of a standardized AE coding system) at 31.6%. Several other key items also demonstrated concerningly low adherence: Item 6a &19 (Reporting of adverse events of special interest) at 37.4%, Item 19 (Explicit reporting of serious AE types) at 39.0%, Item 4a (Identity of the AE assessor(s) at 42.2%, Item 6a (Pre-specification of harm outcomes in the protocol/registration) at 48.1%, and Item 13b and 19 (Discontinuations due to AEs) at 52.9%.

#### 3.2.3 Subgroup analysis: before vs. after guideline publication (temporal trends in item adherence)

Subgroup analysis revealed that following the publication of the CONSORT-Harms 2022 guidelines, most core items showed no statistically significant improvement in adherence rates, with some items even exhibiting a declining trend.

Among the items with decreased adherence, the reduction for Item 4a [Identity of the AE assessor(s)] was statistically significant [RD = −0.193, 95% CI (-0.332,−0.055), *P* = 0.008]. A non-significant downward trend was also observed for Item 6a & 19 (Reporting of adverse events of special interest; RD = −0.100, *P* = 0.156) and Item 14a (Timing of AE occurrence; RD = −0.092, *P* = 0.119).

For items with increased adherence, a positive, though non-significant, trend was observed for Item 6a (Pre-specification of harm outcomes in the protocol/registration; RD = 0.058, *P* = 0.432) and Item 19 (Outcome/Resolution of harms reported; RD = 0.020, *P* = 0.757).

**Figure 4:**
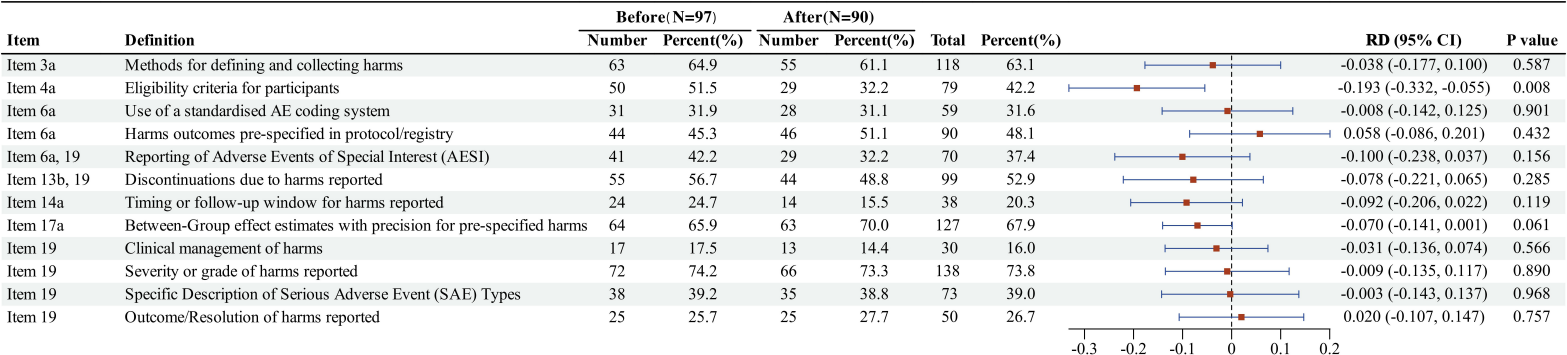
Forest plot of reporting adherence to CONSORT Harms 2022 items before vs. after guideline publication. This forest plot compares the reporting adherence for the 12 AERCI Core items between RCTs published before (2020–2022, *N* = 97) and after (2023–2025, *N* = 90) the release of the CONSORT Harms 2022 guideline.

Overall, this subgroup analysis found no statistically significant difference in the overall quality of adverse event reporting in psoriasis RCTs before vs. after the publication of the CONSORT-Harms 2022 guidelines (Figure 4).

### 3.3 Specific details of AEs

Table 3 details a total of 1,645 adverse events were recorded across the 153 studies that reported specific adverse event types. These events are categorized into 22 distinct classes based on the affected organ systems and etiological mechanisms, each subjected to separate statistical analysis. Diseases of the skin and subcutaneous tissue were the most frequently reported adverse events (360 cases, 21.9%), with pruritus (38 cases) and exacerbation of psoriasis (22 cases) representing the predominant manifestations. Gastrointestinal disorders were the second most common category (236 cases, 14.3%), primarily characterized by diarrhea (56 cases) and nausea (47 cases). Respiratory, thoracic, and mediastinal disorders (233 cases, 14.2%) constituted another frequently reported category, mainly comprising upper respiratory tract infections (59 cases) and nasopharyngitis (58 cases). (When an event was attributable to multiple categories, it was counted only once).

**Table 3:** Specific details of AEs^*^.

**Items**	**AEs**
**Total number of AEs**	**1645**
**Infections and infestations**, ***n*** **(%)**	**47**
Candidiasis	8 (17%)
Candidal infection	3 (6%)
Postoperative wound infection	2 (4%)
Others (Septic shock, etc.)	34 (72%)
**General disorders and administration site conditions**, ***n*** **(%)**	**74**
Fatigue	16 (22%)
Pyrexia	11 (15%)
Dizziness	10 (14%)
Somnolence	5 (7%)
Syncope	4 (5%)
Migraine	3 (4%)
Others (Insomnia, Asthenia, etc.)	25 (34%)
**Gastrointestinal disorders**, ***n*** **(%)**	**236**
Diarrhea	56 (24%)
Nausea	47 (20%)
Vomiting	22 (9%)
Abdominal pain	15 (6%)
Others (Inflammatory bowel disease, Constipation, etc.)	96 (41%)
**Hepatobiliary disorders**, ***n*** **(%)**	**24**
Abnormal liver function	10 (42%)
Cholelithiasis	2 (8%)
Others (Drug-induced liver injury, etc.)	12 (50%)
**Metabolism and nutrition disorders**, ***n*** **(%)**	**42**
Hyperuricemia	9 (21%)
Hyperlipidemia	9 (21%)
Hypertriglyceridemia	6 (14%)
Hypercholesterolemia	4 (10%)
Others (Hyperkalemia, Gout, etc.)	14 (33%)
**Endocrine diseases**, ***n*** **(%)**	**15**
Blood glucose increased	7 (47%)
Goiter	2 (13%)
Others (Hypoglycemia, Primary adrenal insufficiency, etc.)	6 (40%)
**Circulatory disorders**, ***n*** **(%)**	**79**
Hypertension	24 (30%)
Myocardial infarction	5 (6%)
Chest pain	5 (6%)
Angina pectoris	4 (5%)
Others (Palpitations, Sinus bradycardia, etc.)	41 (52%)
**Respiratory, thoracic and mediastinal disorders**, ***n*** **(%)**	**233**
Upper respiratory tract infection	59 (25%)
Nasopharyngitis	58 (25%)
Cough	12 (5%)
Bronchitis	11 (5%)
Others (Pneumonia, Influenza, etc.)	93 (40%)
**Musculoskeletal and connective tissue disorders**, ***n*** **(%)**	**93**
Arthralgia	23 (25%)
Back pain	18 (19%)
Psoriatic arthritis	4 (4%)
Myalgia	4 (4%)
Others (Muscle strain, Fracture, etc.)	44 (47%)
**Injury, poisoning, and procedural complications**, ***n*** **(%)**	**13**
Sunburn	3 (23%)
Ligament sprain	1 (8%)
Scar	1 (8%)
Traumatic hematoma	1 (8%)
Post-vaccination syndrome	1 (8%)
Others	6 (46%)
**Skin and subcutaneous tissue disorders**, ***n*** **(%)**	**360**
Pruritus	38 (11%)
Psoriasis	22 (6%)
Erythema	16 (4%)
Pain	15 (4%)
Eczema	12 (3%)
Folliculitis	12 (3%)
Others (Herpes zoster, Urticaria, etc.)	245 (68%)
**Nervous system disorders**, ***n*** **(%)**	**35**
Paraesthesia	5 (14%)
Tremor	3 (9%)
Ischemic stroke	2 (6%)
Others (Myasthenia, Epilepsy, etc.)	25 (71%)
**Investigations**, ***n*** **(%)**	**128**
Neutropenia	10 (8%)
Lymphocyte count decreased	8 (6%)
Alanine aminotransferase increased	7 (5%)
Aspartate aminotransferase increased	6 (5%)
Hepatic enzyme increased	6 (5%)
Others (WBC decreased, Triglycerides increased, etc.)	91 (71%)
**Others**, ***n*** **(%)**	**56**
Weight decreased	6 (11%)
Weight increased	3 (5%)
Conjunctivitis	3 (5%)
Others (Ocular and ear disorders, etc.)	44 (79%)
**Local reaction at injection site**	**39**
**Psychiatric disorders**	**26**
**Malignant neoplasms**, ***n*** **(%)**	**29**
Melanoma	3 (10%)
Breast cancer	3 (10%)
Basal cell carcinoma	2 (7%)
Others (Liver cancer, Esophageal carcinoma, etc.)	21 (72%)
**Renal and urinary system disorders**, ***n*** **(%)**	**55**
Urinary tract infection	17 (31%)
Urine protein positive	3 (5%)
Others (Hematuria, Proteinuria, etc.)	35 (64%)
**Reproductive and breast disorders**, ***n*** **(%)**	**19**
Pregnancy	4 (21%)
Abortion	3 (16%)
Others (Mastitis, Menorrhagia, etc.)	12 (63%)
**Hematological and lymphatic system disorders**, ***n*** **(%)**	**10**
Sepsis	4 (40%)
Others (Anemia, Osteomyelitis, etc.)	6 (60%)
**Vascular disorders**	**6**
**COVID-19**	**26**

^*^Based on 153 studies that reported specific AE types.

### 3.4 Factors associated with AERCI-Core scores

The results of the univariate and multivariate linear regression analyses are presented in Table 4. In the univariable linear regression analysis, several factors were associated with higher AERCI–Core scores: journal impact factor [β = 0.34, 95% CI (0.21, 0.46), *P* < 0.001], institutional funding [β = 1.57, 95% CI (0.43, 2.71), *P* = 0.007], and sample size (β < 0.01, *P* = 0.001). Based on the multivariate linear regression model adjusting for publication year, sample size, funding source, and disease category, each one-unit increase in journal impact factor[β = 0.27, 95% CI (0.13, 0.40), *P* < .001] and pustular psoriasis [β = 1.85, 95% CI (0.10, 3.60), *P* = .038] with plaque psoriasis as the reference were independently and positively associated with higher adverse event reporting completeness scores. By contrast, publication year [β = −0.18, 95% CI (-0.44, 0.08), *P* = .167], sample size [β = 0.00, 95% CI (-.00,.00), *P* = .101], funding source (including individual, institutional, mixed, and unreported categories; all p>.05), and psoriasis in special areas [β = −0.10, 95% CI (-1.31, 1.11), *P* = .866] did not demonstrate statistically significant associations. Furthermore, the variance inflation factor (VIF) for all independent variables was below 2, indicating the absence of multicollinearity in the model. The variance inflation factors for all variables are detailed in Supplementary Table S2.

**Table 4:** Univariable and multivariable linear regression analyses of factors associated with the AERCI-core score.

**Characteristic**	**N**	**Coefficient (univariable)**	**Coefficient (multivariable)**
Publication year	187	−0.15 (-0.43–0.13, *P* = .286)	−0.18 (-0.44–0.08, *P* = .167)
Funding	187	–	–
None	37	–	–
Individual funding	1	−3.11 (-9.31–3.09, *P* = .324)	−2.82 (-8.67 to 3.03, *P* = .342)
Institutional funding	133	1.57 (0.43–2.71, *P* = .007)	0.42 (-0.77 to 1.61, *P* = .489)
Mixed funding	15	0.09 (-1.78–1.96, *P* = .923)	−0.78 (-2.60 to 1.04, *P* = .398)
Unreported	1	−4.11 (-10.31–2.09, *P* = .193)	−3.81 (-9.67 to 2.04, *P* = .200)
Disease category	187	–	–
Plaque Psoriasis	145	–	–
Pustular Psoriasis	12	1.83 (-0.04–3.71, *P* = .055)	1.85 (0.10–3.60, *P* = .038)
Special area psoriasis	30	−0.57 (-1.81–0.68, *P* = .373)	−0.10 (-1.31–1.11, *P* = .866)
Journal impact factor	187	0.34 (0.21–0.46, *P* < .001)	0.27 (0.13–0.40, *P* < .001)
Sample size	187	0.00 (0.00–0.00, *P* = .001)	0.00 (-0.00–0.00, *P* = .101)

### 3.5 Sensitivity analyses

The results remained robust across all sensitivity analyses. When the AERCI-Core scores were stratified into tertiles and analyzed using ordinal logistic regression, the direction and strength of the associations were consistent with the primary linear regression model. A similar pattern of associations was observed when the analysis was repeated using the extended 20-item index (AERCI-Extended), including the positive correlation between journal impact factor and reporting completeness. Additional analyses conducted after excluding trials with a sample size below 30 did not substantially alter the results (Supplementary Tables S3–S5).

## 4 Discussion

### 4.1 Key findings and interpretation

Guided by the latest CONSORT-Harms (2022) guideline, this study has developed and applied for the first time a structured assessment instrument—AERCI-Core (12 items)—to systematically evaluate the quality of safety reporting in psoriasis RCTs published between 2020 and 2025. The key findings are summarized as follows:

First, our analysis identified significant deficiencies in the overall quality of adverse event reporting among psoriasis RCTs. The AERCI-Core scores (median 6, IQR 2–8, total possible score 0–12) revealed that only approximately one-quarter of studies (26.2%) achieved a high-quality threshold (≥8 points), nearly half (47.1%) were of moderate quality, and over one-quarter (26.7%) were categorized as low quality. Particularly noteworthy is the finding that only three studies (1.6%) reported adherence to all 12 core items. This result aligns with findings by Beytout et al. (14) in dermatology, who used an earlier CONSORT framework and reported a median compliance of 9 checklist items (range 0–18). It is also consistent with the broader literature documenting the generally suboptimal quality of safety reporting across other medical specialties (9, 16–21). These findings collectively reinforce the existence of a gap between reporting guidelines and current practice in this field.

Second, an item-by-item analysis of the AERCI-Core revealed systemic underreporting of critical information. The identity of the adverse event assessor and the method for event confirmation were reported in only 42.2% and 63.1% of trials, respectively, posing challenges for evaluating data objectivity. Information directly pertinent to clinical risk assessment, such as the timing of adverse events (reported in 20.3% of trials), specific management strategies implemented (16.0%), and their subsequent outcomes (26.7%), was documented at alarmingly low rates. Furthermore, reporting rates for the specific types of serious adverse events, adverse events of special interest, and the use of standardized coding systems (e.g., MedDRA) were all below 50% (39.0%, 37.4%, and 31.6%, respectively), substantially impeding the meaningful aggregation and comparison of safety data. The low reporting rates for these specific items represent a substantial deviation from the standards set by CONSORT-Harms 2022 and directly limit the utility of the safety data for informing clinical decision-making.

Further subgroup analysis indicated that, in the period following the publication of the CONSORT-Harms 2022 guideline (2023–2025), compliance rates for most reporting items showed no statistically significant improvement compared to the preceding period (2020–2022). Notably, adherence to several critical items, such as “identity of the adverse event assessor,” exhibited a significant downward trend. This finding suggests that the publication of the guideline did not, by itself, prompt a widespread immediate improvement in reporting practices.

Third, having established the current state of reporting quality and its lack of improvement post-guideline, we further investigated its potential determinants through multivariate regression analysis. The results identified journal impact factor as a key independent predictor of reporting completeness, underscoring the pivotal role of journals in the practical implementation of reporting guidelines. Regression analysis demonstrated that journal impact factor [β = 0.27, 95% CI (0.13, 0.40), *P* < .001] and the pustular psoriasis subtype [β = 1.850, 95% CI (0.10, 3.60), *P* = .038] were independent positive predictors of the AERCI-Core score. It is noteworthy that institutional funding and sample size, which were significant in the univariate analysis, were no longer significant in the multivariate model. This suggests their effects may be mediated through journal impact factor—for example, institutionally funded or larger-scale trials may be more likely to be published in higher-impact journals (22, 23), where stringent editorial and peer-review processes directly promote reporting rigor (24).

This finding, complementary to the results of the subgroup analysis, indicates that the publication of the CONSORT-Harms 2022 guideline has not led to a significant improvement in the quality of adverse event reporting in psoriasis RCTs. On the one hand, subgroup analysis showed no statistically significant increase in compliance rates for most items after the guideline's publication; on the other hand, publication year did not exhibit a significant positive association in the regression model. Taken together, these results consistently indicate that reporting quality has not improved automatically over time or following the introduction of the guideline. Within this context, the independent predictive role of journal impact factor underscores the critical function of journal editorial and peer-review standards in driving the practical adoption of reporting guidelines.

The aforementioned conclusions, of course, should be interpreted with caution. Impact factor is a journal-level metric and does not fully equate to the quality of individual studies; moreover, the analysis may be susceptible to publication bias (25, 26). Future research should further dissect the specific mechanisms by which journals promote reporting quality, focusing on editorial policies, the practical use of manuscript checklists, and the author revision process.

In summary, the AERCI instrument developed in this study provides, for the first time, a structured framework for the quantitative assessment of adverse event reporting quality in RCTs. The assessment clearly identified deficiencies across multiple dimensions of safety reporting in the field of psoriasis, and the release of the CONSORT-Harms 2022 guideline did not significantly alter this landscape in the short term. Although journal impact factor demonstrated a potential positive influence, substantial room for improvement remains in overall reporting quality. Future research efforts, journal editors, and reviewers should place greater emphasis on the implementation of the CONSORT-Harms guidelines, particularly for the key items identified in this study as having low reporting rates. We recommend that journals explicitly mandate adherence to CONSORT-Harms within their author guidelines and systematically verify key safety reporting items during peer review. This approach would translate guideline recommendations into actionable publishing standards, thereby improving the reporting quality of future studies.

### 4.2 Systematic deficiencies at four levels and future directions for improvement

This study identified deficiencies in adverse event reporting within psoriasis RCTs that extend beyond incomplete adherence to the CONSORT-Harms guidelines. These shortcomings permeate four interrelated dimensions—data collection processes, the selection of reported content, and the format of presentation—collectively undermining the reliability of the safety evidence.

#### 4.2.1 Deficiencies in AE data collection and terminology

Our investigation identified non-standardized and incomplete data collection procedures. Despite increased adoption of standardized tools such as MedDRA (27), many studies still employed simplified methods (e.g., frequency tables) for data collection. Over half (57.8%) failed to report AE data collectors. The lack of objective, standardized tools poses considerable challenges to ensuring the comparability and homogeneity of safety data, as well as to the objective evaluation of data collection methods. Furthermore, methodological limitations persist in handling com-plex adverse event data: continuous variables (e.g., blood glucose, blood pressure) are often dichotomized simply as “high/low” (28, 29), or the occurrence of any AE during the entire observation period (e.g., abnormal liver enzymes) is reduced to a binary outcome (“occurred” or “not occurred”) (30). This approach neglects critical details such as event type, frequency, severity, and duration, compromising data integrity and potentially obscuring safety signals or misleading risk assessments. Therefore, we recommend standardizing AE terminology using coding systems such as MedDRA or CTCAE, preserving raw data while avoiding dichotomization (when dichotomization is necessary, distribution-based methods are advised) (31), and pre-specifying reproducible data collection plans (15).

#### 4.2.2 Gaps in outcome selection and reporting completeness

Beyond data collection, deficiencies persist in the selection and completeness of the safety outcomes that are ultimately reported. First, only 73 studies reported specific types of serious adverse events, and only 70 pre-specified adverse events of special interest (AESIs), resulting in insufficient completeness of safety data, a high likelihood of subjective bias, and an inability to extract clinically meaningful information. Therefore, we recommend developing core outcome sets tailored to different therapeutic categories, encompassing core harm outcomes, serious/adverse/unanticipated adverse events (SAE/SAR/SUSAR), investigator-predefined events, and events leading to treatment discontinuation, to systematically prevent omissions of core SAEs and AESIs (32). Selective reporting was evident, with some studies applying arbitrary criteria (e.g., only reporting AEs with >5% incidence) (33) or listing AEs in appendices without meaningful summary in the main text (34, 35). This reporting practice hinders readers' ability to conduct a comprehensive assessment of a treatment's risk profile.

#### 4.2.3 Deficiencies in data presentation and descriptive reporting

First, regarding data presentation, many studies continue to report adverse events in lengthy lists or simplistic frequency tables, which impede readers'ability to quickly discern key risk signals. To address this, we recommend adopting the Safety Planning, Evaluation, and Reporting Team (SPERT) framework to categorize adverse events into three tiers (36). thereby transforming complex safety data into structured, proportional summaries that focus on clinical relevance. We also encourage the use of visual methods (e.g., charts over tables alone) (37), to enhance information clarity and avoid overwhelming the main text with extensive, unstructured lists. Second, descriptive information was frequently lacking. A substantial number of RCTs failed to adequately detail the timing of adverse events, the specific management strategies implemented, and their subsequent outcomes (38–40). This omission hinders the assessment of temporal causality between events and the intervention, prevents evaluation of the clinical management burden, and ultimately diminishes the practical utility of the safety assessment. Future studies should therefore systematically report these core elements.

#### 4.2.4 Limitations in statistical approaches to safety outcomes

Although 67.9% of the studies performed between-group comparisons of safety outcomes, many relied on unadjusted hypothesis testing or solely subjective comparisons of percentage differences in event rates, lacking proper adjustment for confounding variables and the appropriate application of statistical models (41), This analytical approach compromises the validity of the conclusions drawn. In response, we advocate for the adoption of more advanced statistical techniques. More than ten algorithms for adverse drug reaction (ADR) signal detection have been proposed to date, encompassing methods such as Bayesian approaches, multivariate models, and machine learning algorithms (42). Among these, Bayesian methods are particularly well-suited for small-sample scenarios and demonstrate robust overall performance. In psoriasis RCTs, these methods could be employed to identify potential safety signals or to explore associations between adverse events and demographic/clinical characteristics.

### 4.3 Conclusions and implications

This systematic evaluation reveals systemic deficiencies in the current safety reporting of psoriasis RCTs, spanning overall quality, adherence to key checklist items, and multiple facets of data collection, presentation, and analysis. These shortcomings compromise the reliability and clinical utility of the safety evidence. Therefore, future research in this field should not be limited to superficial compliance with reporting checklists (such as CONSORT-Harms) but should strive to establish a fully transparent system encompassing the entire process—from data collection and the selection of core outcomes to data presentation and statistical analysis. Only through such systemic improvement can high–quality safety evidence—sufficient to support robust clinical risk–benefit assessment—be generated.

### 4.4 Limitations

Several limitations of this study should be acknowledged. First, our analysis was restricted to randomized controlled trials published in dermatology specialty journals indexed in the Web of Science. Adverse event reporting practices in general medical journals or journals from other specialties may differ, potentially limiting the generalizability of our findings. Second, adverse event reporting was assessed based on information available in published articles and Supplementary material; safety data documented exclusively in trial protocols, registries, or regulatory submissions may not have been fully captured. Third, although we applied a standardized checklist aligned with the CONSORT-Harms (2022) guideline, the assessment inevitably involved a degree of subjectivity, and the dichotomous scoring approach could not fully capture qualitative differences in reporting depth. Finally, as a cross-sectional meta-epidemiological study, this work is inherently a descriptive analysis of the published literature and cannot establish causal relationships between the observed reporting deficiencies and specific elements of trial design, conduct, or editorial processes. **Fifth, the exclusion of studies with unavailable full texts may have introduced selection bias, as these studies (e.g., those behind paywalls or only published as abstracts) might be systematically different in their reporting completeness. This could potentially lead to an overestimation of the reporting quality in our sample**. Furthermore, the restriction of our inclusion criteria to English-language publications from the past 5 years (2020–2025) may limit the generalizability of our findings to earlier periods or to the non-English literature, precluding an assessment of longitudinal trends in reporting practices.

## Data Availability

The original contributions presented in the study are included in the article/Supplementary material, further inquiries can be directed to the corresponding authors.

## Footnotes

1
https://doi.org/10.17605/OSF.IO/VPT3J
