# Supplementary material for: Reporting of the harms from randomized controlled trials for psoriasis: a cross-sectional meta-epidemiological study
Source: Front Med (Lausanne). 2026 Feb 27;13:1734221. doi: 10.3389/fmed.2026.1734221 (PMC12983368; doi:10.3389/fmed.2026.1734221)
Supplement: Supplementary file 1 [file Supplementary_file_1.docx]

Contents

[Search strategies in database 2](#_Toc20020)

[Journal List 3](#_Toc15754)

[Exclusion after full-text review： 8](#_Toc16822)

[List of Included Studies 13](#_Toc23003)

[Table S1. Adverse Event Reporting Completeness Index (AERCI) Data Extraction Form 40](#_Toc4578)

[Table S2. Multicollinearity Analysis for Each Independent Variable 43](#_Toc23837)

[Table S3. Sensitivity Analysis 1: Multivariable Linear Regression of Factors Associated with AERCI-Core Score After Excluding Trials with Sample Size < 30 44](#_Toc875)

[Table S4. Sensitivity Analysis 2: Multivariable Linear Regression of Factors Associated with the AERCI-Extended Score 45](#_Toc4783)

[Table S5. Sensitivity Analysis 3: Ordinal Logistic Regression of Predictors for AERCI-Core Score Tertiles 46](#_Toc20428)

# Search strategies in database

PUBMED：


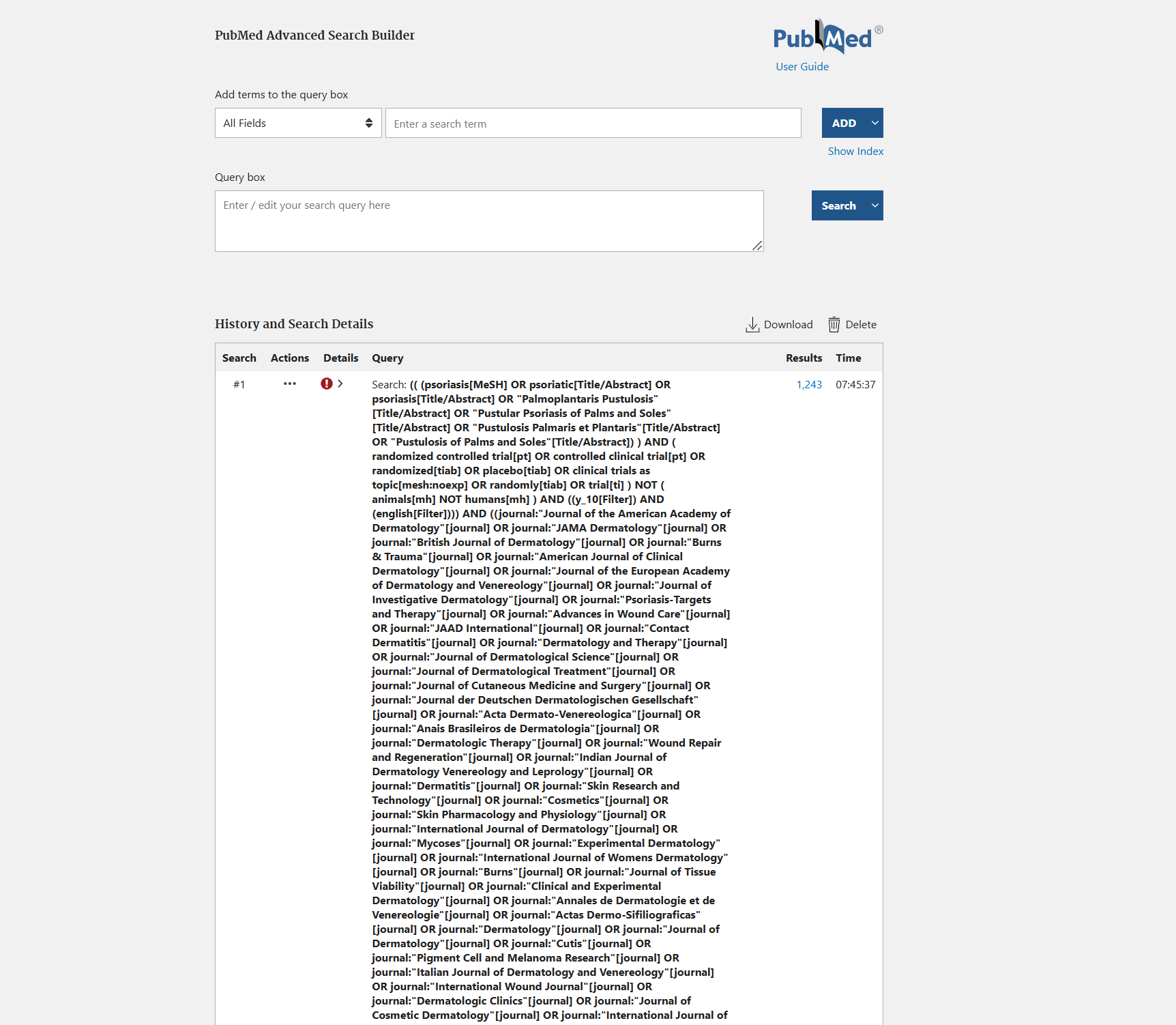


Cochrane Library：


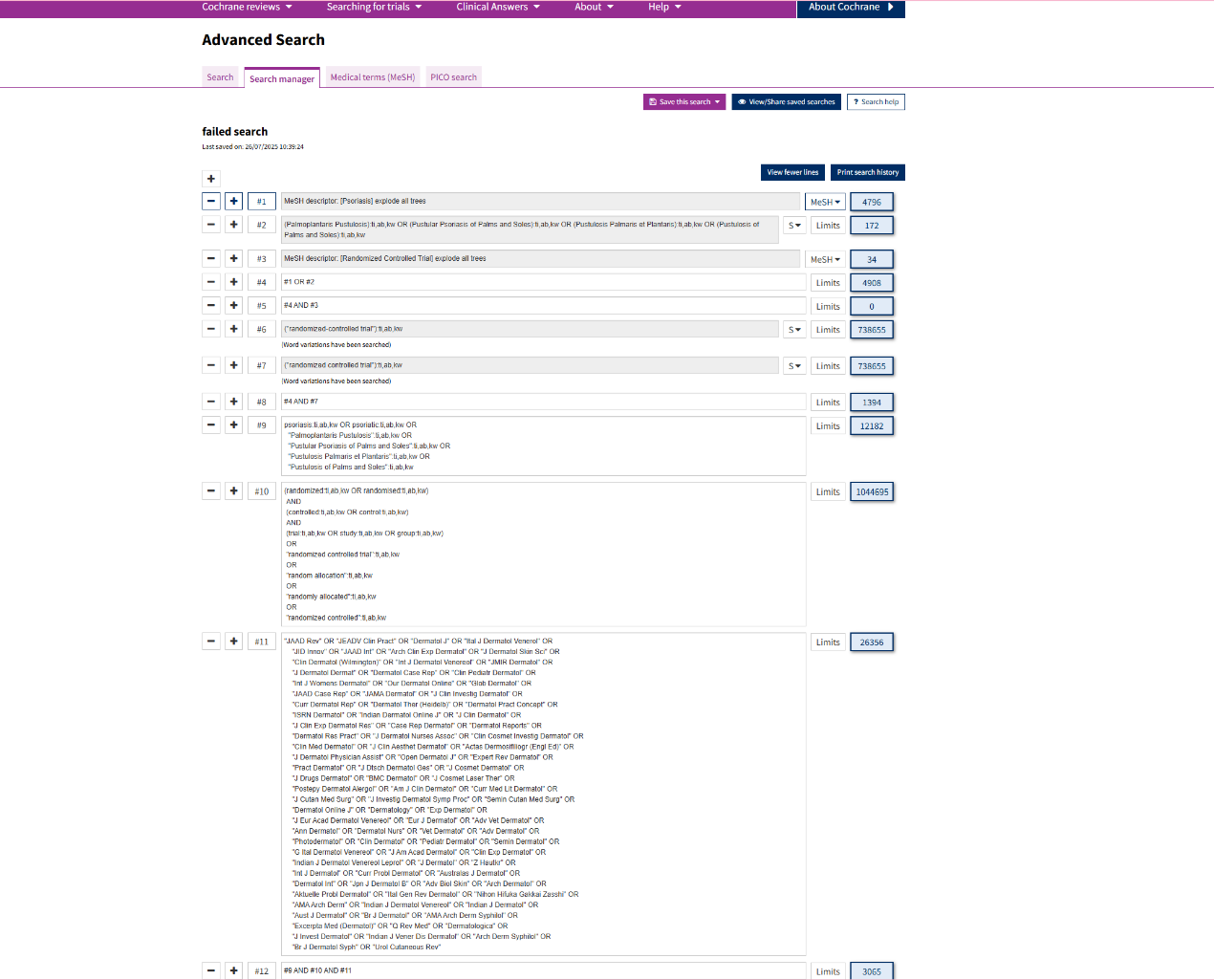


# Journal List

| JOURNAL OF THE AMERICAN ACADEMY OF DERMATOLOGY |
| --- |
| JAMA Dermatology |
| BRITISH JOURNAL OF DERMATOLOGY |
| Burns & Trauma |
| AMERICAN JOURNAL OF CLINICAL DERMATOLOGY |
| JOURNAL OF THE EUROPEAN ACADEMY OF DERMATOLOGY AND VENEREOLOGY |
| JOURNAL OF INVESTIGATIVE DERMATOLOGY |
| Psoriasis-Targets and Therapy |
| Advances in Wound Care |
| JAAD International |
| CONTACT DERMATITIS |
| Dermatology and Therapy |
| JOURNAL OF DERMATOLOGICAL SCIENCE |
| JOURNAL OF DERMATOLOGICAL TREATMENT |
| JOURNAL OF CUTANEOUS MEDICINE AND SURGERY |
| JOURNAL DER DEUTSCHEN DERMATOLOGISCHEN GESELLSCHAFT |
| ACTA DERMATO-VENEREOLOGICA |
| ANAIS BRASILEIROS DE DERMATOLOGIA |
| Dermatitis |
| Dermatologic Therapy |
| WOUND REPAIR AND REGENERATION |
| Indian Journal of Dermatology Venereology & Leprology |
| INTERNATIONAL JOURNAL OF DERMATOLOGY |
| SKIN RESEARCH AND TECHNOLOGY |
| Cosmetics |
| SKIN PHARMACOLOGY AND PHYSIOLOGY |
| MYCOSES |
| EXPERIMENTAL DERMATOLOGY |
| International Journal of Womens Dermatology |
| BURNS |
| CLINICAL AND EXPERIMENTAL DERMATOLOGY |
| Journal of Tissue Viability |
| JOURNAL OF DERMATOLOGY |
| ANNALES DE DERMATOLOGIE ET DE VENEREOLOGIE |
| Actas Dermo-Sifiliograficas |
| DERMATOLOGY |
| CUTIS |
| Pigment Cell & Melanoma Research |
| Italian Journal of Dermatology and Venereology |
| International Wound Journal |
| DERMATOLOGIC CLINICS |
| Journal of Cosmetic Dermatology |
| INTERNATIONAL JOURNAL OF COSMETIC SCIENCE |
| Dermatology Practical & Conceptual |
| CLINICS IN DERMATOLOGY |
| Clinical Cosmetic and Investigational Dermatology |
| PHOTODERMATOLOGY PHOTOIMMUNOLOGY & PHOTOMEDICINE |
| DERMATOLOGIC SURGERY |
| Dermatologica Sinica |
| ARCHIVES OF DERMATOLOGICAL RESEARCH |
| Indian Dermatology Online Journal |
| Journal of Skin Cancer |
| LASERS IN SURGERY AND MEDICINE |
| Dermatology Research and Practice |
| MELANOMA RESEARCH |
| Journal of Drugs in Dermatology |
| AUSTRALASIAN JOURNAL OF DERMATOLOGY |
| Journal of Burn Care & Research |
| Journal of Wound Care |
| Dermatopathology |
| Postepy Dermatologii i Alergologii |
| International Journal of Lower Extremity Wounds |
| EUROPEAN JOURNAL OF DERMATOLOGY |
| Advances in Skin & Wound Care |
| VETERINARY DERMATOLOGY |
| Current Dermatology Reports |
| Journal of Cosmetic and Laser Therapy |
| Annals of Dermatology |
| Skin Appendage Disorders |
| Dermatology Reports |
| INDIAN JOURNAL OF DERMATOLOGY |
| PEDIATRIC DERMATOLOGY |
| European Burn Journal |
| JOURNAL OF CUTANEOUS PATHOLOGY |
| WOUNDS-A COMPENDIUM OF CLINICAL RESEARCH AND PRACTICE |
| AMERICAN JOURNAL OF DERMATOPATHOLOGY |
| Acta Dermatovenerologica Alpina Pannonica et Adriatica |
| Journal of Cutaneous Immunology and Allergy |
| Przeglad Dermatologiczny |
| Case Reports in Dermatology |
| Wound Management & Prevention |
| LEPROSY REVIEW |
| Dermatologie |
| Wound Practice and Research |
| Dermatologie in Beruf und Umwelt |
| JEADV Clinical Practice |
| Acta Dermatovenerologica Croatica |
| Journal of Egyptian Womens Dermatological Society |
| JOURNAL OF COSMETIC SCIENCE |
| Indian Journal of Paediatric Dermatology |
| Journal of the Dermatology Nurses Association |
| Turk Dermatoloji Dergisi-Turkish Journal of Dermatology |
| Turkderm-Turkish Archives of Dermatology and Venerology |
| Egyptian Journal of Dermatology and Venereology |
| Journal of Dermatology & Dermatologic Surgery-JDDS |
| AKTUELLE DERMATOLOGIE |

Source: Clarivate Analytics. (2025). 2025 Journal Citation Reports. Retrieved from the Journal Citation Reports database.

# Exclusion after full-text review：

[1]

Maspero S, Ebert C, Moser S, et al. The potential of instagram to reduce stigmatization of people with psoriasis: a randomized controlled pilot study[J]. Acta Derm Venereol, 2023, 103: adv3513.

[2]

Cestari T F, Souza C D S, Azulay-Abulafia L, et al. Efficacy and safety of risankizumab versus methotrexate in patients with moderate-to-severe plaque psoriasis: results from IMMbrace, a randomized, double-blind, phase 3 study with an open-label extension period in Brazil[J]. An Bras Dermatol, 2025, 100(2): 260-271.

[3]

Foley P, Zhang J, Imafuku S, et al. Deucravacitinib, an oral, selective, allosteric tyrosine kinase 2 inhibitor, in asian patients with moderate to severe psoriasis: findings from the phase 3 POETYK PSO-3 and PSO-4 trials[J]. Australasian Journal of Dermatology, 2023, 64: 28‐29.

[4]

Strober B, Paul C, Blauvelt A, et al. Bimekizumab efficacy and safety through 96 weeks in patients with moderate to severe plaque psoriasis: results from the open-label extension period of the BE RADIANT phase 3b trial[J]. Australasian Journal of Dermatology, 2022, 63(SUPPL 1): 14.

[5]

Muftin Z, Gilbert P, Thompson A R. A randomized controlled feasibility trial of online compassion-focused self-help for psoriasis[J]. Br J Dermatol, 2022, 186(6): 955-962.

[6]

Reich K, Augustin M, Thaçi D, et al. A 24-week multicentre, randomized, open-label, parallel-group study comparing the efficacy and safety of ixekizumab vs. fumaric acid esters and methotrexate in patients with moderate-to-severe plaque psoriasis naive to systemic treatment[J]. Br J Dermatol, 2020, 182(4): 869-879.

[7]

Leutz A, Pinter A, Thaci D, et al. Efficacy and safety of ixekizumab after switching from fumaric acid esters or methotrexate in patients with moderate-to-severe plaque psoriasis naïve to systemic treatment[J]. British Journal of Dermatology, 2021, 184(3): 548‐550.

[8]

Van Voorhees A S, Stein Gold L, Lebwohl M, et al. Efficacy and safety of apremilast in patients with moderate-to-severe plaque psoriasis of the scalp: results up to 32 weeks from a randomized, phase III study[J]. British Journal of Dermatology, 2021, 185(4): 840‐842.

[9]

Tyring S, Moore A, Morita A, et al. Cedirogant in adults with psoriasis: a phase II, randomized, placebo-controlled clinical trial[J]. Clin Exp Dermatol, 2024, 49(11): 1347-1355.

[10]

Johnson M C, Oussedik E, Huang W W, et al. Anecdote increases patient willingness to take a biologic medication for psoriasis[J]. Cutis, 2021, 108(2s): 20-24.

[11]

Okwundu N, Cardwell L A, Cline A E, et al. Adherence to topical treatment can improve treatment-resistant moderate psoriasis[J]. Cutis, 2020, 105(2): 89-91;e2;e3.

[12]

Gisondi P, Bellinato F, Bruni M, et al. Methotrexate vs secukinumab safety in psoriasis patients with metabolic syndrome[J]. Dermatol Ther, 2020, 33(6): e14281.

[13]

Cai L, Li C, Li S, et al. Investigation of the efficacy and safety of xeligekimab (GR1501) in patients with moderate-to-severe plaque psoriasis: a multicenter, randomized, double-blind phase II clinical trial[J]. Dermatol Ther (heidelb), 2025.

[14]

Hrin M L, Bray J K, Feldman S R. Reassurance techniques do not significantly impact confidence in biosimilars for psoriasis: a survey of a convenience sample of individuals with self-identified psoriasis[J]. Dermatol Ther (heidelb), 2022, 12(9): 2173-2180.

[15]

Hrin M L, Feldman S R. Confidence in biosimilar drugs is not much improved by framing them as the “gold” alternative treatment option to bio-originators[J]. Dermatol Ther (heidelb), 2021, 11(4): 1409-1413.

[16]

Morita A, Choon S E, Bachelez H, et al. Design of effisayil^TM^ 2: a randomized, double-blind, placebo-controlled study of spesolimab in preventing flares in patients with generalized pustular psoriasis[J]. Dermatol Ther (heidelb), 2023, 13(1): 347-359.

[17]

Okubo Y, Umezawa Y, Sakurai S, et al. Efficacy and safety of certolizumab pegol in japanese patients with generalized pustular psoriasis and erythrodermic psoriasis: 52-week results[J]. Dermatol Ther (heidelb), 2022, 12(6): 1397-1415.

[18]

Sebastian M, Bagel J, Hoepken B, et al. Single-injection options for administering a 320 mg dose of bimekizumab: 2 mL safety syringe and auto-injector[J]. Dermatol Ther (heidelb), 2025, 15(5): 1113-1134.

[19]

Augustin M, Gottlieb A B, Lebwohl M, et al. Complete skin clearance is associated with the greatest benefits to health-related quality of life and perceived symptoms for patients with psoriasis[J]. Dermatology and Therapy, 2024, 14(10): 2841‐2857.

[20]

Kim J, Lee J, Hawkes J E, et al. Secukinumab improves mild-to-moderate psoriasis: a randomized, placebo-controlled exploratory clinical trial[J]. J Am Acad Dermatol, 2023, 88(2): 428-430.

[21]

Naik H B, Pichard D C, Schwartz D M, et al. Anakinra for refractory pustular psoriasis: a phase II, open-label, dose-escalation trial[J]. J Am Acad Dermatol, 2022, 87(6): 1380-1383.

[22]

Saadi D G, El-Komy M H M, Khedr H, et al. A randomized, controlled pilot study of oral roflumilast compared with intramuscular methotrexate for plaque and scalp psoriasis[J]. J Am Acad Dermatol, 2024, 90(5): 1063-1065.

[23]

Stein Gold L, Adam D N, Albrecht L, et al. Long-term safety and effectiveness of roflumilast cream 0.3% in adults with chronic plaque psoriasis: a 52-week, phase 2, open-label trial[J]. J Am Acad Dermatol, 2024, 91(2): 273-280.

[24]

Imafuku S, Okubo Y, Tada Y, et al. Deucravacitinib, an oral, selective, allosteric tyrosine kinase 2 inhibitor, in japanese patients with moderate to severe plaque, erythrodermic, or generalized pustular psoriasis: efficacy and safety results from an open-label, phase 3 trial[J]. J Dermatol, 2024, 51(3): 365-379.

[25]

Kircik C, Kircik L. Patient preference for calcipotriene and betamethasone dipropionate cream versus foam for the topical treatment of psoriasis: a pilot study[J]. J Drugs Dermatol, 2023, 22(3): 271-273.

[26]

Kircik L H, Draelos Z D, Glick B, et al. Preference for cal/BDP cream or foam in patients with mild to moderate plaque psoriasis[J]. J Drugs Dermatol, 2024, 23(8): 607-611.

[27]

Warren R B, Gold M, Gooderham M, et al. Four-week daily calcipotriene/betamethasone dipropionate foam is highly efficacious in patients with psoriasis (PSO-LONG lead-in phase)[J]. J Drugs Dermatol, 2021, 20(4): 436-441.

[28]

Gelfand J M, Shin D B, Alavi A, et al. A phase IV, randomized, double-blind, placebo-controlled crossover study of the effects of ustekinumab on vascular inflammation in psoriasis (the VIP-U trial)[J]. J Invest Dermatol, 2020, 140(1): 85-93.e2.

[29]

Gelfand J M, Shin D B, Duffin K C, et al. A randomized placebo-controlled trial of secukinumab on aortic vascular inflammation in moderate-to-severe plaque psoriasis (VIP-S)[J]. J Invest Dermatol, 2020, 140(9): 1784-1793.e2.

[30]

Gooderham M J, Mrowietz U, Kadus W, et al. Phase II randomized trial of BI 730357, an oral RORγt inhibitor, for moderate-to-severe plaque psoriasis[J]. J Invest Dermatol, 2025, 145(8): 1969-1978.e14.

[31]

Blauvelt A, Leonardi C L, Gooderham M, et al. Efficacy and safety of continuous risankizumab therapy vs treatment withdrawal in patients with moderate to severe plaque psoriasis: a phase 3 randomized clinical trial[J]. JAMA Dermatol, 2020, 156(6): 649-658.

[32]

Eyerich K, Asadullah K, Pinter A, et al. Noninferiority of 16-week vs 8-week guselkumab dosing in super responders for maintaining control of psoriasis: the GUIDE randomized clinical trial[J]. JAMA Dermatol, 2024, 160(9): 953-963.

[33]

Harris K M, Smilek D E, Byron M, et al. Effect of costimulatory blockade with abatacept after ustekinumab withdrawal in patients with moderate to severe plaque psoriasis: the PAUSE randomized clinical trial[J]. JAMA Dermatol, 2021, 157(11): 1306-1315.

[34]

McMichael A, Shahriari M, Stein Gold L, et al. Guselkumab for moderate to severe scalp psoriasis across all skin tones: cohort B of the VISIBLE randomized clinical trial[J]. JAMA Dermatol, 2025.

[35]

Augustin M, Mrowietz U, Willsmann-Theis D, et al. Ixekizumab versus FAEs and methotrexate in treatment of nail psoriasis in patients with moderate-to-severe psoriasis[J]. Journal Der Deutschen Dermatologischen Gesellschaft [journal of the German Society of Dermatology], 2024, 22(2): 278‐281.

[36]

Tada Y, Armstrong A W, Imafuku S, et al. Deucravacitinib in japanese patients with plaque, generalized pustular, or erythrodermic psoriasis: patient-reported outcomes in the POETYK PSO-4 study[J]. Journal of Dermatology, 2025, 52(2): 353‐358.

[37]

Aichelburg M C, Pinkowicz A, Holzer G, et al. Short- and long-term efficacy of fumaric acid esters or acitretin in combination with a 12-week course of PUVA in the treatment of palmoplantar pustulosis: results from a prospective randomized trial[J]. Journal of the European Academy of Dermatology and Venereology : JEADV, 2021, 35(3): e198‐e200.

[38]

Menter M A, Murakawa G J, Glover H, et al. Clearance of head and neck involvement in plaque psoriasis with tildrakizumab treatment in the phase 3 reSURFACE 1 study[J]. Journal of the European Academy of Dermatology and Venereology : JEADV, 2020, 34(12): e803‐e805.

[39]

Puaratanaarunkon T, Sittisaksomjai S, Sivapornpan N, et al. Topical cannabidiol-based treatment for psoriasis: a dual-centre randomized placebo-controlled study[J]. Journal of the European Academy of Dermatology and Venereology : JEADV, 2022, 36(9): e718‐e720.

[40]

Ryan C, Guenther L, Foley P, et al. Ixekizumab provides persistent improvements in health-related quality of life and the sexual impact associated with moderate-to-severe genital psoriasis in adult patients during a 52-week, randomised, placebo-controlled, phase 3 clinical trial[J]. Journal of the European Academy of Dermatology and Venereology : JEADV, 2022, 36(4): e277‐e279.

[41]

Gyldenløve M, Sørensen J A, Thomsen S F, et al. Improvements in patient-reported outcomes with oral roflumilast for psoriasis: results from a randomized controlled trial (PSORRO)[J]. Journal of the European Academy of Dermatology and Venereology: JEADV, 2025, 39(5): e394‐e397.

# List of Included Studies

[1]

Cai L, Liu H, Mu Z, et al. Safety, pharmacokinetics, and efficiency of JS005, a novel anti-interleukin-17A monoclonal antibody, in healthy chinese adults and patients with moderate to severe psoriasis[J]. Acta Derm Venereol, 2025, 105: adv41105.

[2]

Eckardt M, Stadtmueller L, Zick C, et al. Effects of a brief mindfulness-based intervention in patients with psoriasis: a randomized controlled trial[J]. Acta Derm Venereol, 2024, 104: adv18277.

[3]

Guenther L, Potts Bleakman A, Weisman J, et al. Ixekizumab results in persistent clinical improvement in moderate-to-severe genital psoriasis during a 52 week, randomized, placebo-controlled, phase 3 clinical trial[J]. Acta Derm Venereol, 2020, 100(1): adv00006.

[4]

Renkhold L, Pereira M P, Loser K, et al. Secukinumab reduces psoriasis-associated pruritus and regenerates the cutaneous nerve architecture: results from PSORITUS a doubleblind, placebo-controlled, randomized withdrawal phase IIIb study[J]. Acta Derm Venereol, 2024, 104: adv40737.

[5]

Schultz A, Jensen P, Zibert J R, et al. Recruitment and retention in a randomized control trial of increased dietary protein-to-carbohydrate ratios in patients with psoriasis[J]. Acta Derm Venereol, 2020, 100(1): adv00021.

[6]

Tveit K S, Brokstad K A, Berge R K, et al. A randomized, double-blind, placebo-controlled clinical study to investigate the efficacy of herring roe oil for treatment of psoriasis[J]. Acta Derm Venereol, 2020, 100(10): adv00154.

[7]

Puig L, Lomaga M, Hollister K, et al. An analysis of patient-reported outcomes in ixora-s: comparing ixekizumab and ustekinumab over 52 weeks in moderate-to-severe psoriasis[J]. Acta Dermato-Venereologica, 2020, 100(19): adv00344.

[8]

Kt S, Thakur V, Narang T, et al. Comparison of the efficacy and safety of apremilast and methotrexate in patients with palmoplantar psoriasis: a randomized controlled trial[J]. Am J Clin Dermatol, 2021, 22(3): 415-423.

[9]

Menter A, Cohen S, Kay J, et al. Switching between adalimumab reference product and BI 695501 in patients with chronic plaque psoriasis (VOLTAIRE-X): a randomized controlled trial[J]. Am J Clin Dermatol, 2022, 23(5): 719-728.

[10]

Okubo Y, Kobayashi S, Murakami M, et al. Efficacy and safety of brodalumab, an anti-interleukin-17 receptor a monoclonal antibody, for palmoplantar pustulosis: 16-week results of a randomized clinical trial[J]. Am J Clin Dermatol, 2024, 25(5): 837-847.

[11]

Stein Gold L, Alonso-Llamazares J, Draelos Z D, et al. Effect of roflumilast cream (ARQ-151) on itch and itch-related sleep loss in adults with chronic plaque psoriasis: patient-reported itch outcomes of a phase 2b trial[J]. Am J Clin Dermatol, 2023, 24(2): 305-313.

[12]

Terui T, Okubo Y, Kobayashi S, et al. Efficacy and safety of apremilast for the treatment of japanese patients with palmoplantar pustulosis: results from a phase 2, randomized, placebo-controlled study[J]. Am J Clin Dermatol, 2023, 24(5): 837-847.

[13]

Ju H J, Kim J Y, Jeong D H, et al. Additional use of hyaluronic acid-based dissolving microneedle patches to treat psoriatic plaques: a randomized controlled trial[J]. Ann Dermatol, 2025, 37(2): 105-113.

[14]

Hassan Moftah N, H. Abbas Helmy W, Mohamed Elbakry A, et al. Combined fractional CO2 laser 10,600 nm with methotrexate 1% gel versus methotrexate 1% gel alone in the treatment of nail psoriasis: a randomized comparative study[J]. Arch Dermatol Res, 2024, 317(1): 153.

[15]

Hegazy E M, Taieb M A E, Hassan M H, et al. Plexin B2 tissue expression and related gene polymorphisms in psoriasis and their relation to NB-UVB and acitretin therapy[J]. Arch Dermatol Res, 2024, 316(5): 162.

[16]

Jiang W, Li Q, Zheng W. The impact of biologics targeting the IL-17 and IL-23 pathways on metabolic indicators in plaque psoriasis[J]. Arch Dermatol Res, 2025, 317(1): 643.

[17]

Liu L F, Chen J S, Gu J, et al. Etanercept biosimilar (recombinant human tumor necrosis factor-α receptor II: IgG fc fusion protein) and methotrexate combination therapy in chinese patients with moderate-to-severe plaque psoriasis: a multicentre, randomized, double-blind, placebo-controlled trial[J]. Arch Dermatol Res, 2020, 312(6): 437-445.

[18]

Moftah N H, Elbakry A M, Nouh N M, et al. Excimer light versus topical methotrexate 1% hydrogel in treatment of scalp psoriasis: clinical and dermoscopic study[J]. Arch Dermatol Res, 2025, 317(1): 282.

[19]

Suriano E S, Souza M D M, Kobata C M, et al. Efficacy of an adjuvant lactobacillus rhamnosus formula in improving skin lesions as assessed by PASI in patients with plaque psoriasis from a university-affiliated, tertiary-referral hospital in são paulo (brazil): a parallel, double-blind, randomized clinical trial[J]. Arch Dermatol Res, 2023, 315(6): 1621-1629.

[20]

Andanooru Chandrappa N K, Channakeshavaiah Ravikumar B, Rangegowda S M. Iontophoretic delivery of methotrexate in the treatment of palmar psoriasis: a randomised controlled study[J]. Australas J Dermatol, 2020, 61(2): 140-146.

[21]

Augustin M, Reich K, Yamauchi P, et al. Secukinumab dosing every 2 weeks demonstrated superior efficacy compared with dosing every 4 weeks in patients with psoriasis weighing 90 kg or more: results of a randomized controlled trial[J]. Br J Dermatol, 2022, 186(6): 942-954.

[22]

Blauvelt A, Leonardi C, Elewski B, et al. A head-to-head comparison of ixekizumab vs. guselkumab in patients with moderate-to-severe plaque psoriasis: 24-week efficacy and safety results from a randomized, double-blinded trial[J]. Br J Dermatol, 2021, 184(6): 1047-1058.

[23]

Blauvelt A, Papp K, Gottlieb A, et al. A head-to-head comparison of ixekizumab vs. guselkumab in patients with moderate-to-severe plaque psoriasis: 12-week efficacy, safety and speed of response from a randomized, double-blinded trial[J]. Br J Dermatol, 2020, 182(6): 1348-1358.

[24]

Blauvelt A, Papp K, Trivedi M, et al. Efficacy and safety of the ustekinumab biosimilar ABP 654 in patients with moderate-to-severe plaque psoriasis: a randomized double-blinded active-controlled comparative clinical study over 52 weeks[J]. Br J Dermatol, 2025, 192(5): 826-836.

[25]

Chen J Q, Zheng M, Yin W H, et al. Safety and efficacy of the selective TYK2/JAK1 inhibitor TLL-018 in moderate-to-severe plaque psoriasis: a phase 1b, randomized, double-blind, placebo-controlled study[J]. Br J Dermatol, 2025.

[26]

Cro S, Cornelius V R, Pink A E, et al. Anakinra for palmoplantar pustulosis: results from a randomized, double-blind, multicentre, two-staged, adaptive placebo-controlled trial (APRICOT)[J]. Br J Dermatol, 2021, 186(2): 245-256.

[27]

Gottlieb A B, Kubanov A, van Doorn M, et al. Sustained efficacy of secukinumab in patients with moderate-to-severe palmoplantar psoriasis: 2·5-year results from GESTURE, a randomized, double-blind, placebo-controlled trial[J]. Br J Dermatol, 2020, 182(4): 889-899.

[28]

Hamm H, Wilsmann-Theis D, Tsianakas A, et al. Efficacy and safety of fumaric acid esters in young patients aged 10-17 years with moderate-to-severe plaque psoriasis: a randomized, double-blinded, placebo-controlled trial[J]. Br J Dermatol, 2021, 185(1): 62-73.

[29]

Jiang C, Du Y, Liu X, et al. Safety, tolerability, pharmacokinetics and efficacy of HB0017, a humanized monoclonal antibody that targets interleukin-17A, in healthy participants and patients with moderate-to-severe plaque psoriasis[J]. Br J Dermatol, 2023, 190(1): 28-36.

[30]

Kircik L H, Alonso-Llamazares J, Bhatia N, et al. Once-daily roflumilast foam 0.3% for scalp and body psoriasis: a randomized, double-blind, vehicle-controlled phase IIb study[J]. Br J Dermatol, 2023, 189(4): 392-399.

[31]

Landis M N, Smith S R, Berstein G, et al. Efficacy and safety of topical brepocitinib cream for mild-to-moderate chronic plaque psoriasis: a phase IIb randomized double-blind vehicle-controlled parallel-group study[J]. Br J Dermatol, 2023, 189(1): 33-41.

[32]

Oliver R, Krueger J G, Glatt S, et al. Bimekizumab for the treatment of moderate-to-severe plaque psoriasis: efficacy, safety, pharmacokinetics, pharmacodynamics and transcriptomics from a phase IIa, randomized, double-blind multicentre study[J]. Br J Dermatol, 2022, 186(4): 652-663.

[33]

Reich K, Körber A, Mrowietz U, et al. Secukinumab 2-weekly vs. 4-weekly dosing in patients with plaque-type psoriasis: results from the randomized GAIN study[J]. Br J Dermatol, 2021, 184(5): 849-856.

[34]

Reich K, Puig L, Szepietowski J C, et al. Secukinumab dosing optimization in patients with moderate-to-severe plaque psoriasis: results from the randomized, open-label OPTIMISE study[J]. Br J Dermatol, 2020, 182(2): 304-315.

[35]

Stein Gold L F, Bagel J, Tyring S K, et al. Comparison of risankizumab and apremilast for the treatment of adults with moderate plaque psoriasis eligible for systemic therapy: results from a randomized, open-label, assessor-blinded phase IV study (IMMpulse)[J]. Br J Dermatol, 2023, 189(5): 540-552.

[36]

Thaçi D, Eyerich K, Pinter A, et al. Direct comparison of risankizumab and fumaric acid esters in systemic therapy-naïve patients with moderate-to-severe plaque psoriasis: a randomized controlled trial[J]. Br J Dermatol, 2022, 186(1): 30-39.

[37]

Thaçi D, Pinter A, Sebastian M, et al. Guselkumab is superior to fumaric acid esters in patients with moderate-to-severe plaque psoriasis who are naive to systemic treatment: results from a randomized, active-comparator-controlled phase IIIb trial (POLARIS)[J]. Br J Dermatol, 2020, 183(2): 265-275.

[38]

Warren R B, Barker J, Finlay A Y, et al. Secukinumab for patients failing previous tumour necrosis factor-α inhibitor therapy: results of a randomized open-label study (SIGNATURE)[J]. Br J Dermatol, 2020, 183(1): 60-70.

[39]

Warren R B, Blauvelt A, Poulin Y, et al. Efficacy and safety of risankizumab vs. secukinumab in patients with moderate-to-severe plaque psoriasis (IMMerge): results from a phase III, randomized, open-label, efficacy-assessor-blinded clinical trial[J]. Br J Dermatol, 2021, 184(1): 50-59.

[40]

Zhang J, Ding Y, Wang P, et al. Deucravacitinib, an oral selective allosteric tyrosine kinase 2 inhibitor, in patients from China mainland, taiwan and South Korea with moderate-to-severe plaque psoriasis: a phase III randomized clinical trial[J]. Br J Dermatol, 2025, 192(3): 402-409.

[41]

Berstein G, Zhang Y, Berger Z, et al. A phase I, randomized, double-blind study to assess the safety, tolerability and efficacy of the topical RORC2 inverse agonist PF-06763809 in participants with mild-to-moderate plaque psoriasis[J]. Clin Exp Dermatol, 2021, 46(1): 122-129.

[42]

Bubna A K. Comparison of the clinical efficacy of topical tretinoin 0.05% cream and tacrolimus 0.1% ointment plus iontophoresis in the management of palmoplantar psoriasis[J]. Clin Exp Dermatol, 2024, 49(6): 599-606.

[43]

Choonhakarn C, Chaowattanapanit S, Julanon N, et al. Comparison of the clinical efficacy of subcutaneous vs. oral administration of methotrexate in patients with psoriasis vulgaris: a randomized controlled trial[J]. Clin Exp Dermatol, 2022, 47(5): 942-948.

[44]

El-Komy M H M, Sayed K S, Gawish G, et al. Pulse azathioprine and low-dose methotrexate vs. standard-dose methotrexate in treatment of patients with moderate-to-severe psoriasis: a randomized controlled trial[J]. Clin Exp Dermatol, 2024, 49(9): 1029-1035.

[45]

Svendsen M T, Feldman S R, Mejldal A, et al. Regular support provided by dermatological nurses improves outcomes in patients with psoriasis treated with topical drugs: a randomized controlled trial[J]. Clin Exp Dermatol, 2022, 47(12): 2208-2221.

[46]

Tomalin L E, Kolbinger F, Suprun M, et al. Deep resolution of clinical, cellular and transcriptomic inflammatory markers of psoriasis over 52 weeks of interleukin-17A inhibition by secukinumab[J]. Clin Exp Dermatol, 2024, 49(8): 801-809.

[47]

Yu C, Yu N, Jiang X, et al. Efficacy and safety of supramolecular active zinc in the treatment of scalp psoriasis: a multicentre, randomized, observed-blind, parallel-group, placebo- and active-controlled noninferiority trial[J]. Clin Exp Dermatol, 2023, 48(10): 1138-1144.

[48]

Aboelwafa H O, Abou Khodair Mohamed H, Ibrahim D M, et al. Efficacy of leflunomide compared to methotrexate in the treatment of moderate to severe plaques psoriasis: a randomized controlled clinical trial[J]. Dermatol Pract Concept, 2024, 14(3).

[49]

Hesham Ali Elwan Y, Abdel Azim A, Starace M, et al. Nd:YAG laser in the treatment of nail psoriasis: clinical and dermoscopic assessment[J]. Dermatol Pract Concept, 2021, 11(2): e2021140.

[50]

Akbarzadeh A, Alirezaei P, Doosti-Irani A, et al. The efficacy of lactocare® synbiotic on the clinical symptoms in patients with psoriasis: a randomized, double-blind, placebo-controlled clinical trial[J]. Dermatol Res Pract, 2022, 2022: 4549134.

[51]

Abdelmeniem I M, El Eryan I M, Nofal A, et al. Topical calcipotriol combined with urea 20% versus intralesional injection of triamcinolone acetonide, 5-fluorouracil, and methotrexate in the treatment of nail psoriasis: a comparative study[J]. Dermatol Ther, 2022, 35(9): e15660.

[52]

Al Salman M, Ghiasi M, Farid A S, et al. Oral simvastatin combined with narrowband UVB for the treatment of psoriasis: a randomized controlled trial[J]. Dermatol Ther, 2021, 34(5): e15075.

[53]

Ara S, Mowla M R, Alam M, et al. Efficacy of oral methotrexate (MTX) monotherapy vs oral MTX plus narrowband ultraviolet light B phototherapy in palmoplantar psoriasis[J]. Dermatol Ther, 2020, 33(4): e13486.

[54]

Dogra S, Singh N, Kumar S, et al. Comparison of overall efficacy and safety of oral versus subcutaneous methotrexate in severe psoriasis[J]. Dermatol Ther, 2022, 35(8): e15656.

[55]

Karapetyan S, Davtyan H, Khachikyan K, et al. Impact of supplemental essential phospholipids on treatment outcome and quality of life of patients with psoriasis with moderate severity[J]. Dermatol Ther, 2022, 35(4): e15335.

[56]

Li X, Yang Q, Zheng J, et al. Efficacy and safety of a topical moisturizer containing linoleic acid and ceramide for mild-to-moderate psoriasis vulgaris: a multicenter randomized controlled trial[J]. Dermatol Ther, 2020, 33(6): e14263.

[57]

Ortner V K, Mandel V D, Skak K, et al. Investigating the efficacy and safety of calcipotriol/betamethasone dipropionate foam and laser microporation for psoriatic nail disease-a hybrid trial using a smartphone application, optical coherence tomography, and patient-reported outcome measures[J]. Dermatol Ther, 2022, 35(12): e15965.

[58]

Owczarczyk-Saczonek A, Czerwińska J, Wygonowska E, et al. D-chiro-inositol as a treatment in plaque psoriasis: a randomized placebo-controlled clinical trial[J]. Dermatol Ther, 2021, 34(1): e14538.

[59]

Peng C, Hu Y, Chen W, et al. A randomized prospective study of different dose regimens using the 308-nm excimer laser in the treatment of palmoplantar pustulosis[J]. Dermatol Ther, 2021, 34(5): e15079.

[60]

Shah S, Nikam B, Kale M, et al. Safety and efficacy profile of oral cyclosporine vs oral methotrexate vs oral acitretin in palmoplantar psoriasis: a hospital based prospective investigator blind randomized controlled comparative study[J]. Dermatol Ther, 2021, 34(1): e14650.

[61]

Shatalebi M A, Bokaie Jazi S, Yegdaneh A, et al. Comparative evaluation of gracilaria algae 3% cream vs clobetasol 0.05% cream in treatment of plaque type psoriasis: a randomized, split-body, triple-blinded clinical trial[J]. Dermatol Ther, 2020, 33(6): e14317.

[62]

Sigurgeirsson B, Browning J, Tyring S, et al. Secukinumab demonstrates efficacy, safety, and tolerability upon administration by 2 ml autoinjector in adult patients with plaque psoriasis: 52-week results from MATURE, a randomized, placebo-controlled trial[J]. Dermatol Ther, 2022, 35(3): e15285.

[63]

Armstrong A W, Augustin M, Beaumont J L, et al. Deucravacitinib improves patient-reported outcomes in patients with moderate to severe psoriasis: results from the phase 3 randomized POETYK PSO-1 and PSO-2 trials[J]. Dermatol Ther (heidelb), 2024, 14(8): 2235-2248.

[64]

Bende B, Kui R, Németh A, et al. A randomized controlled trial with a medical device containing sodium hyaluronate and nicotinic acid to increase the efficacy of ultraviolet phototherapy in psoriasis[J]. Dermatol Ther (heidelb), 2020, 10(4): 651-662.

[65]

Burden A D, Bissonnette R, Navarini A A, et al. Spesolimab efficacy and safety in patients with moderate-to-severe palmoplantar pustulosis: a multicentre, double-blind, randomised, placebo-controlled, phase IIb, dose-finding study[J]. Dermatol Ther (heidelb), 2023, 13(10): 2279-2297.

[66]

Catlett I M, Gao L, Hu Y, et al. Pharmacodynamic response to deucravacitinib, an oral, selective, allosteric TYK2 inhibitor, in a global, phase 2, randomized, double-blind, placebo-controlled psoriasis trial[J]. Dermatol Ther (heidelb), 2024, 14(10): 2827-2839.

[67]

Engin B, Güler Özden M, Karstarlı Bakay Ö S, et al. A multicenter randomized double-blind vehicle-controlled parallel group phase 2 study evaluating the efficacy and safety of GN-037 cream in patients with mild-to-moderate plaque psoriasis[J]. Dermatol Ther (heidelb), 2024, 14(12): 3337-3350.

[68]

Jiang C, Zhou H, Zhang W, et al. Efficacy, safety and pharmacokinetics of IL-17 monoclonal antibody injection (AK111) in patients with moderate-to-severe plaque psoriasis: a randomized, double-blinded, placebo-controlled phase ib multidose escalation clinical study[J]. Dermatol Ther (heidelb), 2023, 13(2): 555-567.

[69]

Ludbrook V J, Budd D C, Thorn K, et al. Inhibition of receptor-interacting protein kinase 1 in chronic plaque psoriasis: a multicenter, randomized, double-blind, placebo-controlled study[J]. Dermatol Ther (heidelb), 2024, 14(2): 489-504.

[70]

Mrowietz U, Burden A D, Pinter A, et al. Spesolimab, an anti-interleukin-36 receptor antibody, in patients with palmoplantar pustulosis: results of a phase IIa, multicenter, double-blind, randomized, placebo-controlled pilot study[J]. Dermatol Ther (heidelb), 2021, 11(2): 571-585.

[71]

Odnopozova L, Edin A, Sukharev A, et al. Risankizumab for the treatment of moderate to severe plaque psoriasis in the russian federation[J]. Dermatol Ther (heidelb), 2022, 12(9): 2063-2075.

[72]

Pariser D M, Lebwohl M G, Jaworski J, et al. CT-P17 adalimumab biosimilar in patients with moderate-to-severe chronic plaque psoriasis: an open-label extension of a phase 3 interchangeability study[J]. Dermatol Ther (heidelb), 2025, 15(5): 1079-1092.

[73]

Pinter A, Tsianakas A, Eichner A. Efficacy and safety of topical tacrolimus microemulsion applied twice daily in patients with mild to moderate scalp psoriasis[J]. Dermatol Ther (heidelb), 2024, 14(2): 521-532.

[74]

Reich K, Bianchi L, Khemis A, et al. Brodalumab versus guselkumab in patients with moderate-to-severe psoriasis with an inadequate response to ustekinumab: a randomized, multicenter, double-blind phase 4 trial (COBRA)[J]. Dermatol Ther (heidelb), 2024, 14(2): 453-468.

[75]

Sezer Z, Inal A, Cinar S L, et al. Safety and efficacy of a novel combination cream (GN-037) in healthy volunteers and patients with plaque psoriasis: a phase 1 trial[J]. Dermatol Ther (heidelb), 2023, 13(7): 1489-1501.

[76]

Sidgiddi S, Naqvi S M H, Shenoy M, et al. Efficacy and safety of novel formulation of clobetasol propionate 0.025% cream in indian moderate-to-severe psoriasis patients: phase-2a, randomized 3-arm study[J]. Dermatol Ther (heidelb), 2021, 11(5): 1717-1732.

[77]

Strober B E, Bukhalo M, Armstrong A W, et al. Clinical utility findings of a transcriptomic psoriasis biologic test demonstrate altered physician prescribing behavior and improved patient outcomes[J]. Dermatol Ther (heidelb), 2025, 15(7): 1787-1796.

[78]

Umezawa Y, Sakurai S, Hoshii N, et al. Certolizumab pegol for the treatment of moderate to severe plaque psoriasis: 16-week results from a phase 2/3 japanese study[J]. Dermatol Ther (heidelb), 2021, 11(2): 513-528.

[79]

El Sharkawy D A, El-Komy M H M, Sobhi R M, et al. Fractional CO 2 laser versus fractional CO 2 laser plus betamethasone/calcipotriol ointment in the treatment of nail psoriasis[J]. Dermatologic Surgery, 2023, 49(6): 570‐574.

[80]

Lajevardi V, Kashiri A, Ghiasi M, et al. Evaluating the efficacy of ursodeoxycholic acid plus methotrexate vs methotrexate alone in the treatment of moderate to severe plaque-type psoriasis: a randomized clinical trial[J]. Dermatologic Therapy, 2020, 33(4): e13455.

[81]

Passeron T, Carrascosa J M, Warren R B, et al. A phase IIIb, multicentre, interventional, randomised, placebo-controlled clinical trial investigating the efficacy and safety of guselkumab for the treatment of nonpustular palmoplantar psoriasis (G-PLUS)[J]. Dermatologic Therapy, 2023, 2023.

[82]

Dhaher S A, Mohammed J Q. Etanercept versus methotrexate in the treatment of psoriasis and associated metabolic syndrome: 12-month open-label comparative study[J]. Dermatology, 2024, 240(5-6): 687-693.

[83]

Jendoubi F, Balica S, Richard M A, et al. A multicentre randomised controlled study evaluating the effect of a standardised education programme on quality of life, disease severity, and disease knowledge in patients with moderate-to-severe psoriasis: the EDUPSO study[J]. Dermatology, 2022, 238(4): 630-639.

[84]

Lynch M, Malara A, Timoney I, et al. Sitagliptin and narrow-band ultraviolet-B for moderate psoriasis (DINUP): a randomised controlled clinical trial[J]. Dermatology, 2022, 238(1): 140-147.

[85]

Lynch M, Malara A, Timoney I, et al. Dipeptidyl peptidase-4 inhibition in psoriasis patients with diabetes: a double-blind randomized controlled trial[J]. Dermatology (basel, Switzerland), 2021, 237(1): 66‐69.

[86]

Liu L, Zhang C, Wang J, et al. Comparison of safety and efficacy between calcipotriol plus betamethasone dipropionate gel and calcipotriol scalp solution as long-term treatment for scalp psoriasis in chinese patients: a national, multicentre, prospective, randomized, active-controlled phase 4 trial[J]. Eur J Dermatol, 2020, 30(5): 580-590.

[87]

Heim M, Irondelle M, Duteil L, et al. Impact of topical emollient, steroids alone or combined with calcipotriol, on the immune infiltrate and clinical outcome in psoriasis[J]. Exp Dermatol, 2022, 31(11): 1764-1778.

[88]

Krueger J, Langley R G, Nigen S, et al. Secukinumab versus guselkumab in the complete resolution of ustekinumab-resistant psoriatic plaques: the ARROW study[J]. Exp Dermatol, 2023, 32(10): 1834-1847.

[89]

Sinclair R, Sharifeh S, Thackwray S, et al. Topical application of a novel anti-interleukin-17A antibody fragment penetrates psoriatic skin: results of a randomised, double-blind, placebo-controlled phase ib study[J]. Exp Dermatol, 2023, 32(9): 1538-1545.

[90]

Neema S, Vausdevan B, Misra P, et al. Efficacy of intermittent fasting in the management of chronic plaque psoriasis: a phase IIb clinical trial[J]. Indian Dermatol Online J, 2025, 16(3): 389-396.

[91]

Banerjee S, Das S, Roy A K, et al. Comparative effectiveness and safety of methotrexate versus PUVA in severe chronic stable plaque psoriasis[J]. Indian J Dermatol, 2021, 66(4): 371-377.

[92]

Minh P P T, Minh T T, Thi P H, et al. New-insight UVB treatment for psoriasis vulgaris in vietnamese patients[J]. Indian J Dermatol, 2024, 69(1): 32-37.

[93]

Hassanandani T, Panda M, Jena A K, et al. Methotrexate monotherapy versus methotrexate and apremilast combination therapy in the treatment of palmoplantar psoriasis: a prospective, randomised, assessor-blinded, comparative study[J]. Indian J Dermatol Venereol Leprol, 2023, 89(2): 213-220.

[94]

Morsy E E, Mohamed R, Omar S I. Intense pulsed light versus pulsed dye laser in the treatment of nail psoriasis: intra-patient left to right comparative controlled study[J]. Indian J Dermatol Venereol Leprol, 2024, 90(6): 713-721.

[95]

Singh S K, Singnarpi S R. Safety and efficacy of methotrexate (0.3 mg/kg/week) versus a combination of methotrexate (0.15 mg/kg/week) with cyclosporine (2.5 mg/kg/day) in chronic plaque psoriasis: a randomised non-blinded controlled trial[J]. Indian J Dermatol Venereol Leprol, 2021, 87(2): 214-222.

[96]

Verma K K, Kumar P, Bhari N, et al. Azathioprine weekly pulse versus methotrexate for the treatment of chronic plaque psoriasis: a randomized controlled trial[J]. Indian J Dermatol Venereol Leprol, 2021, 87(4): 509-514.

[97]

Danish M, Khandpur S, Khanna N, et al. A randomized trial comparing 308 nm excimer light with PUVA soak therapy for palmoplantar psoriasis[J]. International Journal of Dermatology, 2025, 64(1): 180‐182.

[98]

Armstrong A W, Gooderham M, Warren R B, et al. Deucravacitinib versus placebo and apremilast in moderate to severe plaque psoriasis: efficacy and safety results from the 52-week, randomized, double-blinded, placebo-controlled phase 3 POETYK PSO-1 trial[J]. J Am Acad Dermatol, 2023, 88(1): 29-39.

[99]

Blauvelt A, Arenberger P, Sauder M B, et al. Highly selective, allosteric inhibition of TYK2 with oral ESK-001 in patients with moderate-to-severe plaque psoriasis: results from STRIDE, a 12-week, randomized, double-blinded, placebo-controlled, dose-ranging phase 2 study[J]. J Am Acad Dermatol, 2025.

[100]

Elewski B E, Lebwohl M G, Anadkat M J, et al. Rapid and sustained improvements in generalized pustular psoriasis physician global assessment scores with spesolimab for treatment of generalized pustular psoriasis flares in the randomized, placebo-controlled effisayil 1 study[J]. J Am Acad Dermatol, 2023, 89(1): 36-44.

[101]

Feldman S R, Narbutt J, Girolomoni G, et al. A randomized, double-blind, phase III study assessing clinical similarity of SB17 (proposed ustekinumab biosimilar) to reference ustekinumab in subjects with moderate-to-severe plaque psoriasis[J]. J Am Acad Dermatol, 2024, 91(3): 440-447.

[102]

Fiorillo L, Becker E, de Lucas R, et al. Efficacy and safety of apremilast in pediatric patients with moderate-to-severe plaque psoriasis: 16-week results from SPROUT, a randomized controlled trial[J]. J Am Acad Dermatol, 2024, 90(6): 1232-1239.

[103]

Gebauer K, Spelman L, Yamauchi P S, et al. Efficacy and safety of tildrakizumab for the treatment of moderate-to-severe plaque psoriasis of the scalp: a multicenter, randomized, double-blind, placebo-controlled, phase 3b study[J]. J Am Acad Dermatol, 2024, 91(1): 91-99.

[104]

Hashim P W, Chima M, Kim H J, et al. Crisaborole 2% ointment for the treatment of intertriginous, anogenital, and facial psoriasis: a double-blind, randomized, vehicle-controlled trial[J]. J Am Acad Dermatol, 2020, 82(2): 360-365.

[105]

Lebwohl M, Bukhalo M, Stein Gold L, et al. A randomized phase 3b study evaluating the safety and efficacy of risankizumab in adult patients with moderate-to-severe plaque psoriasis with non-pustular palmoplantar involvement[J]. J Am Acad Dermatol, 2024, 91(6): 1150-1157.

[106]

Lebwohl M, Kircik L, Lacour J P, et al. Twice-weekly topical calcipotriene/betamethasone dipropionate foam as proactive management of plaque psoriasis increases time in remission and is well tolerated over 52 weeks (PSO-LONG trial)[J]. J Am Acad Dermatol, 2021, 84(5): 1269-1277.

[107]

Magnolo N, Kingo K, Laquer V, et al. A phase 3 open-label, randomized multicenter study to evaluate efficacy and safety of secukinumab in pediatric patients with moderate to severe plaque psoriasis: 24-week results[J]. J Am Acad Dermatol, 2022, 86(1): 122-130.

[108]

Man X, Zaharieva K, Pulka G, et al. A randomized phase III study to compare efficacy and safety of BAT2206 (proposed ustekinumab biosimilar) with reference ustekinumab in patients with moderate to severe plaque psoriasis[J]. J Am Acad Dermatol, 2025, 92(4): 724-731.

[109]

Merola J F, Parish L C, Guenther L, et al. Efficacy and safety of apremilast in patients with moderate-to-severe genital psoriasis: results from DISCREET, a phase 3 randomized, double-blind, placebo-controlled trial[J]. J Am Acad Dermatol, 2024, 90(3): 485-493.

[110]

Pariser D M, Bagel J, Lebwohl M, et al. Serlopitant for psoriatic pruritus: a phase 2 randomized, double-blind, placebo-controlled clinical trial[J]. J Am Acad Dermatol, 2020, 82(6): 1314-1320.

[111]

Rousel J, Bergmans M E, van der Meulen L W J, et al. Guselkumab induction therapy demonstrates long-lasting efficacy in patients with mild psoriasis, results from a randomized, placebo-controlled exploratory clinical trial[J]. J Am Acad Dermatol, 2024, 90(2): 395-397.

[112]

Sofen H L, Gebauer K, Spelman L, et al. Efficacy and safety of tildrakizumab for the treatment of moderate-to-severe plaque psoriasis of the scalp: week 52 results from a phase 3b, randomized, double-blind, placebo-controlled trial[J]. J Am Acad Dermatol, 2025, 92(4): 816-824.

[113]

Stein Gold L, Bhatia N, Tallman A M, et al. A phase 2b, randomized clinical trial of tapinarof cream for the treatment of plaque psoriasis: secondary efficacy and patient-reported outcomes[J]. J Am Acad Dermatol, 2021, 84(3): 624-631.

[114]

Stein Gold L, Papp K, Pariser D, et al. Efficacy and safety of apremilast in patients with mild-to-moderate plaque psoriasis: results of a phase 3, multicenter, randomized, double-blind, placebo-controlled trial[J]. J Am Acad Dermatol, 2022, 86(1): 77-85.

[115]

Strober B, Thaçi D, Sofen H, et al. Deucravacitinib versus placebo and apremilast in moderate to severe plaque psoriasis: efficacy and safety results from the 52-week, randomized, double-blinded, phase 3 program fOr evaluation of TYK2 inhibitor psoriasis second trial[J]. J Am Acad Dermatol, 2023, 88(1): 40-51.

[116]

Tehlirian C, Singh R S P, Pradhan V, et al. Oral tyrosine kinase 2 inhibitor PF-06826647 demonstrates efficacy and an acceptable safety profile in participants with moderate-to-severe plaque psoriasis in a phase 2b, randomized, double-blind, placebo-controlled study[J]. J Am Acad Dermatol, 2022, 87(2): 333-342.

[117]

Van Voorhees A S, Stein Gold L, Lebwohl M, et al. Efficacy and safety of apremilast in patients with moderate to severe plaque psoriasis of the scalp: results of a phase 3b, multicenter, randomized, placebo-controlled, double-blind study[J]. J Am Acad Dermatol, 2020, 83(1): 96-103.

[118]

Warren R B, French L E, Blauvelt A, et al. Orismilast in moderate-to-severe psoriasis: efficacy and safety from a 16-week, randomized, double-blinded, placebo-controlled, dose-finding, and phase 2b trial (IASOS)[J]. J Am Acad Dermatol, 2024, 90(3): 494-503.

[119]

Xia L, Miao G, Yang X, et al. Efficacy and safety of SYSA1902 versus reference ustekinumab in moderate-to-severe plaque psoriasis: a multicenter, randomized, phase III study[J]. J Am Acad Dermatol, 2025, 93(1): 115-123.

[120]

Yan K, Li F, Bi X, et al. Efficacy and safety of vunakizumab in moderate-to-severe chronic plaque psoriasis: a randomized, double-blind, placebo-controlled phase 3 trial[J]. J Am Acad Dermatol, 2025, 92(1): 92-99.

[121]

Young P M, Chen A Y, Ford A R, et al. Effects of online care on functional and psychological outcomes in patients with psoriasis: a randomized controlled trial[J]. J Am Acad Dermatol, 2023, 88(2): 364-370.

[122]

Yu Y, Yi X, Zhang Y, et al. Comparison of different incremental dose regimens of narrow-band ultraviolet B in skin types III to V: a prospective, randomized, single-blind, parallel study in patients with psoriasis[J]. J Am Acad Dermatol, 2021, 84(4): 1136-1138.

[123]

Zhang C, Yan K, Diao Q, et al. A multicenter, randomized, double-blinded, placebo-controlled, dose-ranging study evaluating the efficacy and safety of vunakizumab in patients with moderate-to-severe plaque psoriasis[J]. J Am Acad Dermatol, 2022, 87(1): 95-102.

[124]

Essa Abd Elazim N, Mahmoud Abdelsalam A, Mohamed Awad S. Efficacy of combined fractional carbon dioxide laser and topical tazarotene in nail psoriasis treatment: a randomized intrapatient left-to-right study[J]. J Cosmet Dermatol, 2022, 21(7): 2808-2816.

[125]

Hegazy E M, El Taieb M A, Ibrahim H M, et al. TWEAK levels in psoriatic patients treated with narrowband ultraviolet B and methotrexate[J]. J Cosmet Dermatol, 2024, 23(5): 1905-1911.

[126]

Helmy A Y, El Batreek M H, Abdel Fadeel D A, et al. Efficacy of the topical cyclosporine cream assisted by fractional carbon dioxide laser vs topical clobetasol cream for the treatment of plaque psoriasis: randomized comparative study[J]. J Cosmet Dermatol, 2022, 21(8): 3362-3370.

[127]

Massiot P, Pinto P C, Leclerc-Mercier S, et al. Clinical benefit and tolerance profile of a keratolytic and hydrating shampoo in subjects with mild to moderate psoriasis. Results from a double-blind, randomized, vehicle-controlled study[J]. J Cosmet Dermatol, 2023, 22(7): 2050-2053.

[128]

Chen H, Sun J, Yang H, et al. Fixed combination of tazarotene and betamethasone dipropionate for treatment of psoriasis vulgaris: the result of a phase 3, multicenter, randomized controlled trial[J]. J Dermatol, 2020, 47(7): 728-734.

[129]

Morita A, Yamaguchi Y, Tateishi C, et al. Efficacy and safety of apremilast and phototherapy versus phototherapy only in psoriasis vulgaris[J]. J Dermatol, 2022, 49(12): 1211-1220.

[130]

Okubo Y, Morishima H, Zheng R, et al. Sustained efficacy and safety of guselkumab in patients with palmoplantar pustulosis through 1.5 years in a randomized phase 3 study[J]. J Dermatol, 2021, 48(12): 1838-1853.

[131]

Okubo Y, Murakami M, Kobayashi S, et al. Risankizumab in japanese patients with moderate-to-severe palmoplantar pustulosis: results from the randomized, phase 3 JumPPP study[J]. J Dermatol, 2025, 52(4): 593-602.

[132]

Youn S W, Jo S J, Park C J, et al. Bimekizumab efficacy and safety in korean patients with moderate to severe plaque psoriasis: a phase 3, randomized, placebo-controlled, double-blinded study[J]. J Dermatol, 2024, 51(11): 1392-1403.

[133]

Zhang J, Ding Y, Wang P, et al. Deucravacitinib, an oral, selective, allosteric tyrosine kinase 2 inhibitor, in asian patients with moderate to severe psoriasis: improvements in patient-reported outcomes in a randomized trial[J]. J Dermatol, 2025.

[134]

Blauvelt A, Pariser D M, Tyring S, et al. Psoriasis improvements and inflammatory biomarker normalization with secukinumab: the randomized ObePso-S study[J]. J Dermatol Sci, 2023, 109(1): 12-21.

[135]

Atalay S, Berends S E, Groenewoud H M M, et al. Serum drug levels and anti-drug antibodies in the context of dose tapering by interval prolongation of adalimumab, etanercept and ustekinumab in psoriasis patients: results of the CONDOR trial[J]. J Dermatolog Treat, 2022, 33(5): 2680-2684.

[136]

Blauvelt A, Gordon K B, Lee P, et al. Efficacy, safety, usability, and acceptability of risankizumab 150 mg formulation administered by prefilled syringe or by an autoinjector for moderate to severe plaque psoriasis[J]. J Dermatolog Treat, 2022, 33(4): 2085-2093.

[137]

Cline A, Unrue E L, Cardwell L A, et al. Internet-based survey intervention improves adherence to methotrexate among psoriasis patients[J]. J Dermatolog Treat, 2022, 33(6): 2784-2789.

[138]

El-Hanafy G M, El-Komy M H M, Nashaat M A, et al. The impact of methotrexate therapy with vitamin D supplementation on the cardiovascular risk factors among patients with psoriasis; a prospective randomized comparative study[J]. J Dermatolog Treat, 2022, 33(3): 1617-1622.

[139]

Feldman S R, Narbutt J, Girolomoni G, et al. Biosimilar SB17 versus reference ustekinumab in moderate to severe plaque psoriasis after switching: phase 3 study results up to week 52[J]. J Dermatolog Treat, 2024, 35(1): 2436607.

[140]

Ferris L K, Ott E, Jiang J, et al. Efficacy and safety of guselkumab, administered with a novel patient-controlled injector (one-press), for moderate-to-severe psoriasis: results from the phase 3 ORION study[J]. J Dermatolog Treat, 2020, 31(2): 152-159.

[141]

Khashaba S A, Gamil H, Salah R, et al. Efficacy of long-pulsed Nd-YAG laser in the treatment of nail psoriasis: a clinical and dermoscopic evaluation[J]. J Dermatolog Treat, 2021, 32(4): 446-452.

[142]

Lin L, Xu X, Yu Y, et al. Glucagon-like peptide-1 receptor agonist liraglutide therapy for psoriasis patients with type 2 diabetes: a randomized-controlled trial[J]. J Dermatolog Treat, 2022, 33(3): 1428-1434.

[143]

Okwundu N, Cardwell L, Cline A, et al. Is topical treatment effective for psoriasis in patients who failed topical treatment?[J]. J Dermatolog Treat, 2021, 32(1): 41-44.

[144]

Sigurgeirsson B, Schäkel K, Hong C H, et al. Efficacy, tolerability, patient usability, and satisfaction with a 2 mL pre-filled syringe containing secukinumab 300 mg in patients with moderate to severe plaque psoriasis: results from the phase 3 randomized, double-blind, placebo-controlled ALLURE study[J]. J Dermatolog Treat, 2022, 33(3): 1718-1726.

[145]

Holmback J, Carlsson A, Rinwa P. Efficacy and safety of water-free lipid formulation system containing calcipotriol against psoriasis vulgaris[J]. J Drugs Dermatol, 2023, 22(2): 197-202.

[146]

Kircik L H, Schlesinger T E, Tanghetti E. Efficacy and safety of calcipotriene 0.005%/betamethasone dipropionate 0.064% foam with apremilast for moderate plaque psoriasis[J]. J Drugs Dermatol, 2020, 19(9): 874-880.

[147]

Kircik L, Jacobson A. Halobetasol propionate 0.01% and tazarotene 0.045% lotion with a ceramide-containing moisturizer in adults with psoriasis[J]. J Drugs Dermatol, 2024, 23(2): 50-53.

[148]

Liu J, Cices A, Kaufman B, et al. Efficacy and safety of calcipotriene/betamethasone dipropionate foam in the treatment of psoriasis in skin of color[J]. J Drugs Dermatol, 2023, 22(2): 165-173.

[149]

Moludi J, Fathollahi P, Khedmatgozar H, et al. Probiotics supplementation improves quality of life, clinical symptoms, and inflammatory status in patients with psoriasis[J]. J Drugs Dermatol, 2022, 21(6): 637-644.

[150]

Nestor M S, Fischer D, Arnold D. Randomized, investigator-blinded study to compare the efficacy and tolerance of a 650-microsecond, 1064-nm YAG laser to a 308-nm excimer laser for the treatment of mild to moderate psoriasis vulgaris[J]. J Drugs Dermatol, 2020, 19(2): 176-183.

[151]

Papp K A, Gooderham M, Droege M, et al. Roflumilast cream improves signs and symptoms of plaque psoriasis: results from a phase 1/2a randomized, controlled study[J]. J Drugs Dermatol, 2020, 19(8): 734-740.

[152]

Stein Gold L, Green L J, Dhawan S, et al. A phase 3, randomized trial demonstrating the improved efficacy and patient acceptability of fixed dose calcipotriene and betamethasone dipropionate cream[J]. J Drugs Dermatol, 2021, 20(4): 420-425.

[153]

Bagel J, Blauvelt A, Nia J, et al. Secukinumab maintains superiority over ustekinumab in clearing skin and improving quality of life in patients with moderate to severe plaque psoriasis: 52-week results from a double-blind phase 3b trial (CLARITY)[J]. J Eur Acad Dermatol Venereol, 2021, 35(1): 135-142.

[154]

Bodemer C, Kaszuba A, Kingo K, et al. Secukinumab demonstrates high efficacy and a favourable safety profile in paediatric patients with severe chronic plaque psoriasis: 52-week results from a phase 3 double-blind randomized, controlled trial[J]. J Eur Acad Dermatol Venereol, 2021, 35(4): 938-947.

[155]

Holzer G, Hoke M, Sabeti-Sandor S, et al. Disparate effects of adalimumab and fumaric acid esters on cardiovascular risk factors in psoriasis patients: results from a prospective, randomized, observer-blinded head-to-head trial[J]. J Eur Acad Dermatol Venereol, 2021, 35(2): 441-449.

[156]

Iversen L, Conrad C, Eidsmo L, et al. Secukinumab demonstrates superiority over narrow-band ultraviolet B phototherapy in new-onset moderate to severe plaque psoriasis patients: week 52 results from the STEPIn study[J]. J Eur Acad Dermatol Venereol, 2023, 37(5): 1004-1016.

[157]

Kaul M, Jarvis P, Rozenberg I, et al. First-in-human study demonstrating the safety and clinical efficacy of novel anti-IL-17A monoclonal antibody CJM112 in moderate to severe plaque psoriasis[J]. J Eur Acad Dermatol Venereol, 2021, 35(5): 1143-1151.

[158]

Mrowietz U, Barker J, Conrad C, et al. Efficacy and safety of apremilast in patients with limited skin involvement, plaque psoriasis in special areas and impaired quality of life: results from the EMBRACE randomized trial[J]. J Eur Acad Dermatol Venereol, 2023, 37(2): 348-355.

[159]

Navarini A A, Prinz J C, Morita A, et al. Spesolimab improves patient-reported outcomes in patients with generalized pustular psoriasis: results from the effisayil 1 study[J]. J Eur Acad Dermatol Venereol, 2023, 37(4): 730-736.

[160]

Papp K A, Armstrong A W, Koresawa T, et al. Efficacy and safety of ME3183, an oral phosphodiesterase 4 inhibitor, in patients with plaque psoriasis[J]. J Eur Acad Dermatol Venereol, 2025.

[161]

Papp K A, Beyska-Rizova S, Gantcheva M L, et al. Efficacy and safety of piclidenoson in plaque psoriasis: results from a randomized phase 3 clinical trial (COMFORT-1)[J]. J Eur Acad Dermatol Venereol, 2024, 38(6): 1112-1120.

[162]

Pinter A, Hoffmann M, Reich K, et al. A phase 4, randomized, head-to-head trial comparing the efficacy of subcutaneous injections of brodalumab to oral administrations of fumaric acid esters in adults with moderate-to-severe plaque psoriasis (CHANGE)[J]. J Eur Acad Dermatol Venereol, 2021, 35(3): 701-711.

[163]

Pinter A, Reich A, Arenberger P, et al. Randomized phase 3 trial demonstrating high efficacy, favourable safety and convenience of a novel calcipotriol and betamethasone dipropionate cream for the treatment of psoriasis[J]. J Eur Acad Dermatol Venereol, 2023, 37(11): 2327-2335.

[164]

Thaçi D, Soliman A M, Eyerich K, et al. Patient-reported outcomes with risankizumab versus fumaric acid esters in systemic therapy-naïve patients with moderate to severe plaque psoriasis: a phase 3 clinical trial[J]. J Eur Acad Dermatol Venereol, 2021, 35(8): 1686-1691.

[165]

Warren R B, Strober B, Silverberg J I, et al. Oral orismilast: efficacy and safety in moderate-to-severe psoriasis and development of modified release tablets[J]. J Eur Acad Dermatol Venereol, 2023, 37(4): 711-720.

[166]

Yélamos O, Alejo B, Ertekin S S, et al. Non-invasive clinical and microscopic evaluation of the response to treatment with clobetasol cream vs. calcipotriol/betamethasone dipropionate foam in mild to moderate plaque psoriasis: an investigator-initiated, phase IV, unicentric, open, randomized clinical trial[J]. J Eur Acad Dermatol Venereol, 2021, 35(1): 143-149.

[167]

Forman S B, Pariser D M, Poulin Y, et al. TYK2/JAK1 inhibitor PF-06700841 in patients with plaque psoriasis: phase IIa, randomized, double-blind, placebo-controlled trial[J]. J Invest Dermatol, 2020, 140(12): 2359-2370.e5.

[168]

Garshick M S, Drenkova K, Barrett T J, et al. A randomized open-label clinical trial of lipid-lowering therapy in psoriasis to reduce vascular endothelial inflammation[J]. J Invest Dermatol, 2022, 142(6): 1749-1752.e4.

[169]

Page K M, Suarez-Farinas M, Suprun M, et al. Molecular and cellular responses to the TYK2/JAK1 inhibitor PF-06700841 reveal reduction of skin inflammation in plaque psoriasis[J]. J Invest Dermatol, 2020, 140(8): 1546-1555.e4.

[170]

van der Kraaij G, Busard C, van den Reek J, et al. Adalimumab with methotrexate vs. Adalimumab monotherapy in psoriasis: first-year results of a single-blind randomized controlled trial[J]. J Invest Dermatol, 2022, 142(9): 2375-2383.e6.

[171]

Juntongjin P, Srisinlapakig S, Nitayavardhana S. Botulinum toxin injection shows promise in nail psoriasis: a comparative randomized controlled trial[J]. JAAD Int, 2024, 16: 105-111.

[172]

Alexis A, McMichael A, Soung J, et al. Guselkumab for moderate to severe psoriasis across all skin tones: cohort a of the VISIBLE randomized clinical trial[J]. JAMA Dermatol, 2025.

[173]

Armstrong A W, Gooderham M, Lynde C, et al. Tyrosine kinase 2 inhibition with zasocitinib (TAK-279) in psoriasis: a randomized clinical trial[J]. JAMA Dermatol, 2024, 160(10): 1066-1074.

[174]

Atalay S, van den Reek J, den Broeder A A, et al. Comparison of tightly controlled dose reduction of biologics with usual care for patients with psoriasis: a randomized clinical trial[J]. JAMA Dermatol, 2020, 156(4): 393-400.

[175]

Gelfand J M, Armstrong A W, Lim H W, et al. Home- vs office-based narrowband UV-B phototherapy for patients with psoriasis: the LITE randomized clinical trial[J]. JAMA Dermatol, 2024, 160(12): 1320-1328.

[176]

Gooderham M J, Alonso-Llamazares J, Bagel J, et al. Roflumilast foam, 0.3%, for psoriasis of the scalp and body: the ARRECTOR phase 3 randomized clinical trial[J]. JAMA Dermatol, 2025, 161(7): 698-706.

[177]

Jenssen M, Furberg A S, Jorde R, et al. Effect of vitamin D supplementation on psoriasis severity in patients with lower-range serum 25-hydroxyvitamin D levels: a randomized clinical trial[J]. JAMA Dermatol, 2023, 159(5): 518-525.

[178]

Li X, Li B, Yang D, et al. Safety and efficacy of anti-IL-23 monoclonal antibody QX004N for patients with psoriasis: a randomized clinical trial[J]. JAMA Dermatol, 2025, 161(3): 247-255.

[179]

Tull T, Cro S, Barker J, et al. 052 anakinra for palmoplantar pustulosis: results from a randomized, double-blind, multicentre, two staged, adaptive placebo controlled trial (APRICOT)[J]. Journal of Investigative Dermatology, 2021, 141(10): S157.

[180]

Cerminara S E, Cvijetic I, Huber S, et al. ApreScalp: a phase 4 multicentre, randomized, placebo-controlled study evaluating the effect of apremilast on pruritus and quality of life of patients with moderate-to-severe scalp psoriasis[J]. Journal of the European Academy of Dermatology and Venereology: JEADV, 2025, 39(5): e424‐e426.

[181]

Chen Y, Yi M, Pang X, et al. Effects of secukinumab combined with tretinoin on metabolism, liver enzymes, and inflammatory factors in patients with moderate to severe psoriasis vulgaris[J]. Postepy Dermatol Alergol, 2024, 41(1): 113-120.

[182]

AlMutairi N, Eassa B I. Comparing the efficacy and safety of IL-17 inhibitors for treatment of moderate-to-severe psoriasis: a randomized double blind pilot study with a review of literature[J]. Postepy Dermatologii I Alergologii, 2021, 38(2): 281‐288.

[183]

Dall’Oglio F, Verzì A E, Guglielmi G, et al. A new prescription emollient device (PED) for psoriasis of sensitive areas and folds: a randomized prospective open trial[J]. Psoriasis (auckl), 2024, 14: 135-142.

[184]

Hölsken S, Krefting F, Mühlhaus S, et al. Shaping treatment expectation to optimize efficacy of interleukin 17A antagonist secukinumab in psoriasis patients[J]. Psoriasis (auckl), 2025, 15: 9-22.

[185]

Viswanath V, Joshi P, Lawate P, et al. An open-label, randomized, prospective, comparative, three-arm clinical trial to evaluate the safety and effectiveness of apremilast with three different titration methods in patients with chronic plaque psoriasis in India[J]. Psoriasis (auckl), 2022, 12: 53-61.

[186]

Lademann J, Mansouri P, Nahavandi A, et al. In vivo skin penetration, radical protection, and structural changes after topical application of a herbal oil cream compared to topical calcipotriol in mild to moderate psoriasis[J]. Skin Pharmacol Physiol, 2021, 34(6): 337-350.

[187]

Mohammadi F, Harofteh F Z, Sahebnasagh A, et al. Efficacy and safety of topical rosuvastatin & melatonin vs. placebo in patients with mild to moderate plaque psoriasis: a preliminary randomized double-blinded clinical trial[J]. Skin Res Technol, 2024, 30(4): e13689.

# **Table S1.** Adverse Event Reporting Completeness Index (AERCI) Data Extraction Form

| No. | CONSORT-Harms (2022) item | CONSORT-Harms Item Description | AERCI-Core Corresponding Component | Score（0/1） |
| --- | --- | --- | --- | --- |
| 1 | Item 3a | How harms were identified (methods and definitions) | Definition and method of adverse event ascertainment | ☐0 ☐1 |
| 2 | Item 4a | Eligibility criteria for participants | AE assessor identity | ☐0 ☐1 |
| 3 | Item 6a | Use of a coding system for harms (e.g., MedDRA, WHO-ART) | MedDRA / WHO-ART used | ☐0 ☐1 |
| 4 | Item 6a | Harm outcomes pre-specified in protocol/registry | Registry/protocol mention | ☐0 ☐1 |
| 5 | Item 6a, 19 | Definition and reporting of adverse events of special interest | AESI specified | ☐0 ☐1 |
| 6 | Item 13b, 19 | Participants withdrawn or lost to follow-up due to harms | AE-related withdrawal | ☐0 ☐1 |
| 7 | Item 14a | When harms were collected and the duration of follow-up | Time of onset reported | ☐0 ☐1 |
| 8 | Item 17a | Statistical methods used to compare harms between intervention groups | Between-group AE analysis | ☐0 ☐1 |
| 9 | Item 19 | How harms were managed | AE management described | ☐0 ☐1 |
| 10 | Item 19 | Severity grading of harms | AE severity reported | ☐0 ☐1 |
| 11 | Item 19 | Numbers and types of serious adverse events | SAE types specified | ☐0 ☐1 |
| 12 | Item 19 | Outcomes of harms (e.g., resolved, ongoing, sequelae) | AE outcome reported | ☐0 ☐1 |
| 13 | Item 1b | Structured abstract with harms results and conclusions | The structured abstract must contain dedicated results and conclusions regarding harms. | ☐0 ☐1 |
| 14 | Item 6c | Description of how non-prespecified harms here identified | If applicable, describe whether non-prespecified benefit-risk outcomes were identified, specify the screening criteria, and detail how non-prespecified harms were identified (e.g., by reviewing laboratory data, spontaneous reports). | ☐0 ☐1 |
| 15 | Item 12a | Pre-specified statistical methods for comparing harms between groups | Pre-specify the statistical methods used for comparing harms between groups (e.g, risk difference is recommended). | ☐0 ☐1 |
| 16 | Item 13a,16 | Analysis population for harms (safety set) clearly defined | Clearly indicate, in the flow diagram or text, that the population analyzed for harms is the "safety set" (participants who received at least one dose of the intervention), not necessarily the intention-to-treat set. | ☐0 ☐1 |
| 17 | Item 17a | Between-Group effect estimates with precision for pre-specified harms | For each pre-specified harm outcome, provide between-group effect estimates and their precision (e.g., 95% confidence interval). | ☐0 ☐1 |
| 18 | Item 17b | Presentation of both absolute and relative risks | Strongly encourage the presentation of both absolute risks (e.g., per 1000 person-years) and relative risks for harms. | ☐0 ☐1 |
| 19 | Item 18 | Reporting of other analyses (subgroup/adjusted) and their pre-specification | Report results of any other analyses (e.g., subgroup, adjusted) for harms, indicating which were pre-specified and which were exploratory. | ☐0 ☐1 |
| 20 | Item 24,17a | Protocol Access or Explanation for Unreported Safety Data | Provide information on how to access the full trial protocol (which includes the detailed harms assessment plan) or explain any unreported safety data. | ☐0 ☐1 |
|  |  | Total AERCI Score | 0–12 points (sum of item scores) |  |

*Note Scoring: 1 = adequately reported; 0 = not reported or unclear.

# **Table S2.** Multicollinearity Analysis for Each Independent Variable

| Variable | GVIF | Df | GVIF^(1/(2*Df)) | Tolerance |
| --- | --- | --- | --- | --- |
| Publication year | 1.021 | 1.000 | 1.010 | 0.979 |
| Funding | 1.299 | 4.000 | 1.033 | 0.770 |
| Disease category | 1.138 | 2.000 | 1.033 | 0.878 |
| Journal impact factor | 1.263 | 1.000 | 1.124 | 0.792 |
| Sample size | 1.181 | 1.000 | 1.087 | 0.847 |

# **Table S3.** Sensitivity Analysis 1: Multivariable Linear Regression of Factors Associated with AERCI-Core Score After Excluding Trials with Sample Size < 30

| Variable | β Coefficient | Standard Error | t value | p value |
| --- | --- | --- | --- | --- |
| (Intercept) | 945.549 | 632.348 | 1.495 | 0.137 |
| Publication year | -0.464 | 0.313 | -1.482 | 0.140 |
| Funding source (Reference: None) | | | | 0.322* |
| Individual | -6.998 | 7.048 | -0.993 | 0.322 |
| Institutional | 1.068 | 1.432 | 0.746 | 0.457 |
| Mixed | -1.368 | 2.193 | -0.624 | 0.534 |
| Unreported | -8.883 | 7.051 | -1.260 | 0.209 |
| Disease subtype (Reference: Plaque Psoriasis) | | | | 0.027* |
| Pustular Psoriasis | 4.682 | 2.108 | 2.222 | 0.028 |
| Special area psoriasis | 0.151 | 1.457 | 0.104 | 0.917 |
| Journal impact factor | 0.630 | 0.161 | 3.910 | 0.000 |
| Sample size | 0.003 | 0.002 | 1.619 | 0.107 |

*Abbreviation: AERCI-Core, Adverse Event Reporting Completeness Index–Core.

*Note: AERCI-Core, Adverse Event Reporting Completeness Index–Core. This sensitivity analysis excluded 21 trials with a sample size below 30 participants. The outcome variable was the continuous AERCI-Core score (range: 0-12). The p-values marked with an asterisk (*) for categorical variables represent the overall significance of the variable from the likelihood ratio test.

# **Table S4.** Sensitivity Analysis 2: Multivariable Linear Regression of Factors Associated with the AERCI-Extended Score

| Variable | β Coefficient (Estimate) | Standard Error | t value | p value |
| --- | --- | --- | --- | --- |
| (Intercept) | 550.445 | 285.137 | 1.930 | 0.055 |
| Publication year | -0.271 | 0.141 | -1.920 | 0.057 |
| Funding source (Reference: None) | | | | |
| Individual | -2.976 | 2.989 | -0.996 | 0.321 |
| Institutional | 0.526 | 0.664 | 0.792 | 0.430 |
| Mixed | -1.118 | 1.037 | -1.078 | 0.283 |
| Unreported | -3.724 | 2.986 | -1.247 | 0.214 |
| Disease subtype (Reference: Plaque Psoriasis) | | | | |
| Pustular Psoriasis | 1.730 | 0.899 | 1.925 | 0.056 |
| Special area psoriasis | -0.217 | 0.696 | -0.312 | 0.755 |
| Journal impact factor | 0.281 | 0.073 | 3.846 | ＜0.001 |
| Sample size | 0.001 | 0.001 | 1.238 | 0.218 |

*Abbreviation: AERCI-Extended, Adverse Event Reporting Completeness Index–Extended (score range: 0–20).

*Note:This sensitivity analysis used the extended 20-item index as the outcome variable to assess whether the factors associated with reporting completeness differed when using a more comprehensive assessment tool.

# **Table S5.** Sensitivity Analysis 3: Ordinal Logistic Regression of Predictors for AERCI-Core Score Tertiles

| Variable | Value | Standard Error | t value | LR Chisq | Df | Pr(>Chisq) |
| --- | --- | --- | --- | --- | --- | --- |
| Publication year | -0.062 | 0.000 | -351.050 | 0.539 | 1.000 | 0.463 |
| Funding source (Reference: None) | | | | | | |
| Individual | -15.417 | 0.000 | -878980643.616 | 4.213 | 4.000 | 0.378 |
| Institutional | 0.043 | 0.303 | 0.142 |  |  |  |
| Mixed | -0.515 | 0.256 | -2.009 |  |  |  |
| Unreported | -15.517 | 0.000 | -844649368.913 |  |  |  |
| Disease subtype (Reference: Plaque Psoriasis) | | | | | | |
| Pustular Psoriasis | 1.336 | 0.539 | 2.481 | 5.701 | 2.000 | 0.058 |
| Special area psoriasis | -0.186 | 0.372 | -0.500 |  |  |  |
| Journal impact factor | 0.145 | 0.046 | 3.180 | 10.309 | 1.000 | 0.001 |
| Sample size | 0.001 | 0.001 | 1.538 | 2.581 | 1.000 | 0.108 |

*Note:This table presents results from an ordered logistic regression (proportional odds model) using the polr function in R, with the AERCI-Core reporting quality score categorized into tertiles (Low: ≤33rd percentile; Medium: 33rd–66th percentile; High: >66th percentile).
